# Luteolin targets MKK4 to attenuate particulate matter-induced MMP-1 and inflammation in human keratinocytes

**DOI:** 10.1038/s41598-025-01090-3

**Published:** 2025-05-15

**Authors:** Jaehyeok Yun, Jong-Eun Kim

**Affiliations:** Department of Food Science and Technology, Korea National University of Transportation, Jeungpyeong, Republic of Korea

**Keywords:** Biochemistry, Cell biology, Chemical biology, Environmental sciences, Health care, Molecular medicine

## Abstract

Particulate matter (PM) is an environmental pollutant that causes premature skin aging and inflammation. In the present study, we investigated the protective effects of luteolin, a bioactive flavonoid, against PM-induced skin damage in human keratinocytes (HaCaT cells). We examined the antioxidant and anti-inflammatory properties of luteolin, focusing on its ability to inhibit key markers of skin aging and inflammation, specifically matrix metalloproteinase-1 (MMP-1) and cyclooxygenase-2 (COX-2). The results demonstrated that luteolin effectively suppressed PM-induced MMP-1 and COX-2 expression and reduced the production of the proinflammatory cytokine IL-6. Mechanistically, luteolin inhibited the activation of AP-1 and NF-κB pathways and decreased reactive oxygen species (ROS) levels in HaCaT cells. Additionally, luteolin binds directly to mitogen-activated protein kinase kinase (MKK) 4, inhibiting its kinase activity and subsequently reducing the phosphorylation of JNK1/2 and p38 mitogen-activated protein kinase. These findings suggest that luteolin can mitigate PM-induced skin aging and inflammation through multiple molecular pathways, highlighting its potential as a therapeutic agent to protect skin health.

**Supplementary Information:**

The online version contains supplementary material available at 10.1038/s41598-025-01090-3.

## Introduction

Particulate matter (PM), comprising dust particles suspended in the air, includes both naturally occurring dust and particles formed by anthropogenic activities^1^. PM is composed of nitrates, sulfates, elemental and organic carbon, organic compounds such as polycyclic aromatic hydrocarbons, biological components such as endotoxins and cell fragments, and metals such as iron, copper, nickel, zinc, and vanadium^2,3^. Sources of PM include industrial activities, vehicular emissions, and coal-fired power plants. PM also has significant health impacts^4^. Owing to its small size, PM can penetrate the human respiratory system, leading to the exacerbation of respiratory and cardiovascular diseases^5,6^. PM is recognized as a more serious health threat than other common air pollutants such as ozone and carbon monoxide^3^.

PM exposure causes premature skin aging, inflammation, irritation, and hyperpigmentation^7^. These particles penetrate the epidermal barrier and trigger oxidative stress and inflammatory responses that degrade collagen and elastin, which are essential for skin elasticity and firmness^8^. This results in redness, itching, and exacerbation of conditions, such as eczema and acne, along with increased melanin production, leading to dark spots and an uneven skin tone^9^. PM affects skin health by interfering with the cellular signal transduction pathways. Oxidative stress from PM generates reactive oxygen species (ROS), activating pathways such as NF-κB, which regulate proinflammatory cytokines^10^. The mitogen-activated protein kinase (MAPK) pathway responds to stress and inflammation, leading to the expression of inflammatory cytokines and matrix metalloproteinases (MMPs) that degrade collagen. Understanding these mechanisms emphasizes the importance of protecting the skin from PM to maintain health and prevent related disorders^7^.

**Fig. 1:**
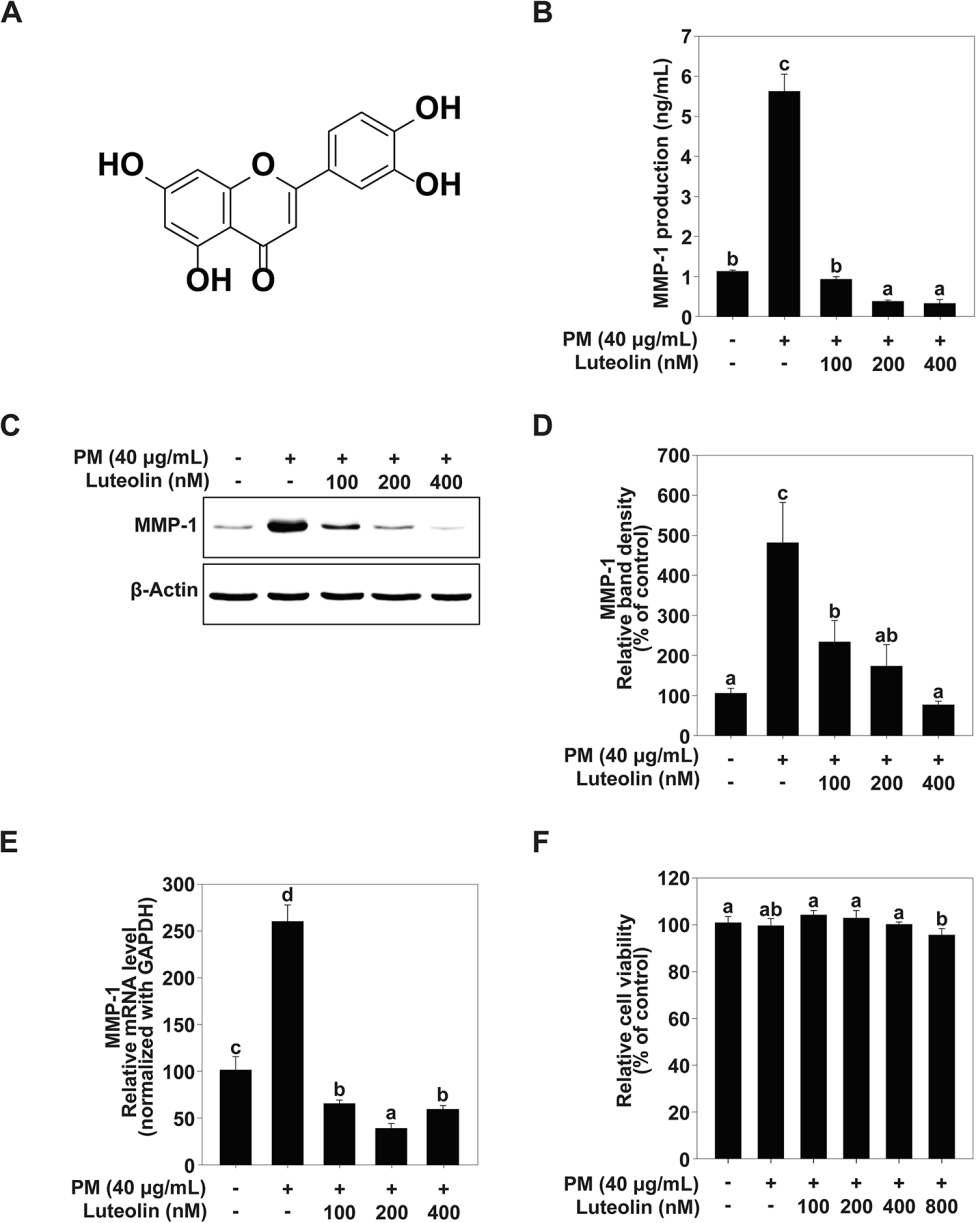
Effect of luteolin on PM-induced MMP-1 production and expression. (**A**) Chemical structure of luteolin. (**B**) Effect of luteolin on matrix metalloproteinase (MMP)-1 production in human keratinocytes (HaCaT) cells. HaCaT cells were pretreated with luteolin at the indicated concentrations for 1 h, followed by exposure to PM (40 µg/mL). After 24 h, the culture media were collected, and MMP-1 production was measured using ELISA assay kits. (**C**, **D**) Effect of luteolin on MMP-1 expression in HaCaT cells. HaCaT cells were pretreated with luteolin at the indicated concentrations for 1 h, followed by exposure to particulate matter (PM) (40 µg/mL). After 24 h, the cells were lysed as described in the Materials and Methods section. MMP-1 protein levels were analyzed using western blotting. β-Actin was detected to verify equal loading of proteins. Images were captured using FUSION Solo S (VILVER Lourmat., Paris, FRA). (**E**) Effect of luteolin on MMP-1 mRNA expression in HaCaT cells. MMP-1 mRNA levels were analyzed using quantitative real-time PCR. HaCaT cells were pretreated with luteolin at the indicated concentrations for 1 h, followed by exposure to PM (40 µg/mL). After 24 h, the cells were lysed as described in the Materials and Methods section. Data (*n* = 3) are shown as the mean ± SD. (**F**) Effect of luteolin on HaCaT cell viability. HaCaT cells were pretreated with luteolin at the indicated concentrations for 1 h, followed by exposure to PM (40 µg/mL). After 24 h, cell viability was measured using an MTT assay. Data (*n* = 5) are shown as the mean ± SD. The mean with letters (a–d) on the graph indicates a significant difference (*p* < 0.05) in one-way ANOVA, and Duncan’s Multiple Range test was used as a post-test.

Luteolin (Fig. 1A) is a bioactive compound with positive effects on skin health^11^. Research indicates that luteolin is effective in preventing skin damage and aging caused by ultraviolet (UV) radiation, owing to its antioxidant and anti-inflammatory properties^12^. For example, luteolin can reduce the adverse photobiological effects of UVA and UVB radiation, acting as the first line of defense and modulating inflammatory processes in the skin^11^. Additionally, luteolin exerts significant chemopreventive effects^13,14^. It also helps alleviate the symptoms of inflammatory skin diseases such as psoriasis and atopic dermatitis by suppressing the expression of proinflammatory cytokines^15^. Furthermore, luteolin reduced UVA-induced autophagy in skin cells, thereby enhancing cell survival^16^.

MMPs are endopeptidases that contain zinc and possess a wide array of substrate specificities. Together, these enzymes can break down diverse elements of extracellular matrix (ECM) proteins^17^. MMP-1, among MMPs, plays a crucial role in skin aging by degrading collagen types I and III. Overexpression of MMP-1 breaks down collagen, which constitutes 90% of the dermis, resulting in decreased skin elasticity and wrinkle formation, ultimately contributing to skin aging^18–21^. PM increases the expression of MMP-1 in the skin, and inhibiting MMP-1 serves as a preventive strategy against skin aging^22,23^. Cyclooxygenases (COXs) are responsible for initiating and controlling the initial step of the transformation of arachidonic acid into prostaglandins (PGs)^24^. There are two primary isoforms of COX, COX-1 and COX-2. COX-1 is constitutively expressed in most tissues and its expression remains relatively constant. In contrast, COX-2 expression can be significantly increased by various mitogenic and inflammatory triggers such as growth factors, cytokines, hormones, bacterial endotoxins, tumor promoters, and UV light^24^. COX-2 converts arachidonic acid to prostaglandin E_2_ (PGE_2_). An increase in PGE_2_ levels can negatively affect inflammatory responses associated with chronic inflammation, skin cancer, and other skin-related inflammatory conditions^25–27^. Increased COX-2 expression is induced by PM. Inhibiting the expression of COX-2 could serve as an effective strategy to suppress skin inflammation^28^. PM-induced expression of MMP-1 and COX-2 is associated with the MAPK signaling pathway^23,29^. The MAPK signaling pathway is a crucial signaling pathway that regulates proliferation, differentiation, apoptosis, and stress responses^30^. Controlling this MAPK signaling pathway could serve as an excellent strategy for inhibiting MMP-1 and COX-2 expression induced by PM.

Considering the detrimental effects of PM on skin health, there is an urgent need to explore protective strategies. Despite advancements in the understanding of the impact of PM on skin aging and inflammation, effective interventions remain limited. Owing to its potent antioxidant and anti-inflammatory properties, luteolin has emerged as a promising candidate for mitigating PM-induced skin damage. By targeting key pathways such as NF-κB and MAPK, luteolin may offer comprehensive protection against oxidative stress and inflammatory responses triggered by PM. Therefore, further investigation into the mechanisms by which luteolin counteracts PM-induced skin damage is crucial. This study aimed to fill this gap by elucidating the protective effects of luteolin on PM-induced skin alterations, providing a scientific basis for the development of novel skin care formulations and therapeutic strategies. The effects of luteolin on MMP-1 and COX-2 induced by PM were studied using HaCaT cells, and the mechanism was investigated.

## Materials and methods

### Chemicals and materials

Luteolin was purchased from Sigma-Aldrich (St. Louis, MO, USA). Standardized fine dust (PM10-like) (European Reference Material ERM-CZ100) was purchased from the European Commission’s Joint Research Centre (Brussels, Belgium). Dulbecco’s modified Eagle’s medium (DMEM), penicillin-streptomycin solution, and trypsin-EDTA solution were purchased from Welgene (Gyeongsan, Korea). Ascorbic acid was acquired from LPS solution (Daejeon, Korea). Fetal bovine serum (FBS) was obtained from Atlas Biologicals (Fort Collins, CO, USA). The MMP-1 antibody was purchased from R&D Systems Inc. (Minneapolis, MN, USA). Antibodies against total MKK 4, total MKK 3/6, total MEK 1/2, p38 mitogen-activated protein kinases, extracellular signal-regulated kinase (ERK) 1/2 (Thr202/Tyr204), c-Jun N-terminal kinase (JNK) 1/2, p90RSK (Thr359/Ser363), and β-actin were purchased from Santa Cruz Biotechnology (Dallas, TX, USA). Other antibodies were acquired from Cell Signaling Biotechnology (Danvers, MA, USA). The 3-[4,5-dimethylthiazol-2-yl]-2,5-diphenyltetrazolium bromide (MTT) powder was purchased from USB Co. (Cleveland, OH). 2′,7′-dichlorofluorescin-diacetate was obtained from Sigma-Aldrich (St. Louis, MO, USA). Lentiviral expression vectors including pGF-AP1-mCMV-EF1-Puro, pGF-NF-κB-mCMV-EF1-Puro, and pGF-MMP-1-mCMV-EF1-puro (System Biosciences, CA, USA), along with packaging vectors including pMD2.0G and psPAX, were purchased from Addgene Inc. (Cambridge, MA, USA). ECL Prime western blotting Detection Reagent was procured from Amersham (Little Chalfont, UK).

### Cell culture

HaCaT keratinocytes were obtained from CLS Cell Lines Services GmbH (Heidelberg, Germany) and cultured at 37 °C, 85–95% humidity with 5% CO_2_ in growth medium (10% FBS-DMEM) supplemented with 1% antibiotics. Cells were maintained by subculturing at 80–90% confluence.

### Preparation of particulate matter

PM was prepared at a concentration of 25 mg/mL, mixed with dimethyl sulfoxide (DMSO), and sonicated for 40 min to prevent agglomeration. The PM stock solution was diluted with serum-free DMEM for use in experiments.

### Cell viability

Cell viability was assessed using the MTT assay. HaCaT cells were cultured in 96-well plates, incubated in serum-free DMEM for 24 h, treated with luteolin for 1 h, and treated with PM. After 24 h of treatment, the cells were exposed to the MTT solution (0.45 mg/mL) and incubated at 37 °C with 5% CO_2_ for 30 min. The medium was removed, and the formazan dye in the cells was solubilized in 100 µL of DMSO. The absorbance was measured at 517 nm using a microplate reader (BioTek, VT, USA).

### Western blot

HaCaT cells were incubated in serum-free DMEM for 24 h, treated with luteolin (100, 200, or 400 nM) for 1 h, and then treated with PM. Cell lysates were prepared using Cell Lysis Buffer [20 mM Tris-HCl (pH 7.5), 150 mM NaCl, 1 mM Na_2_EDTA, 1 mM EGTA, 1% Triton, 2.5 mM sodium pyrophosphate, 1 mM beta-glycerophosphate, 1 mM Na_3_VO_4_, 1 µg/mL] (Cell Signaling, Beverly, MA, USA). Cell lysates were harvested on ice, centrifuged at 12,970 rpm for 10 min, and the supernatants were collected. The protein concentrations were determined using a protein assay kit (Bio-Rad, Hercules, CA, USA). The proteins were separated using electrophoresis on 6% and 10% SDS-polyacrylamide gels and transferred onto PVDF membranes (Amersham, UK). The membrane was blocked with 5% non-fat milk or 5% BSA for 1 h, followed by incubation with specific primary antibodies overnight at 4 °C. After washing, an HRP-conjugated secondary antibody (Gendepot, Texas, USA) was applied and incubated at room temperature for 1 h. All primary antibodies were used at a dilution of 1:1000, and secondary antibodies were also used at 1:5000. Protein bands were visualized using ECL Prime western blotting detection reagent. Western blot data were quantitatively analyzed using an image analysis system (Vilber Lourmat, Marne La Vallee, France).

### Enzyme-linked immunosorbent assay

The amounts of Human MMP-1, PGE_2_ and IL-6 released into the medium were measured using Human MMP-1, PGE_2_ and IL-6 ELISA kits (R&D Systems Inc., Minneapolis, MN, USA) following the manufacturer’s instructions.

### Quantitative real-time PCR

RNAs was extracted from HaCaT cells using RNAiso Plus (Takara Bio, Inc., Shiga, Japan). The RNA concentration and purity were measured using Take3 (Biotek, VT, USA). Reverse transcription was performed using a PrimeScript™ 1st Strand cDNA Synthesis Kit (Takara Bio Inc., Japan). RT-PCR was conducted with TB Green^®^ Premix Ex Taq™ (Tli RNaseH Plus). cDNA samples were run in triplicate, with glyceraldehyde 3-phosphate dehydrogenase (GAPDH) as the internal control. The following primers were used: COX-2 forward (5′- GCA GTT GTT CCA GAC AAG CA -3′), COX-2 reverse (5′- CAG GAT ACA GCT CCA CAG CA -3′); MMP-1 forward (5′- CCC CAA AAG CGT GTG ACA GTA − 3′), MMP-1 reverse (5′- GGT AGA AGG GAT TTG TGC G -3′); IL-6 forward (5′- CAA TCT GGA TTC AAT GAG GAG AG -3′), IL-6 reverse (5′- CTC TGG CTT GTT CCT CAC TAC TC -3′); GAPDH forward (5′- GAG TCA ACG GAT TTG GTC GT -3′), GAPDH reverse (5′- TTG ATT TTG GAG GGA TCT CG -3′).

### ROS measurement

ROS levels were measured using the DCF-DA assay. HaCaT cells were cultured in 96-well black plates and incubated in serum-free DMEM for 24 h. Cells were then exposed to DCF-DA solution (100 µM) for 30 min, washed with Hank’s Balanced Salt Solution (HBSS), and incubated for an additional 30 min. After removing the HBSS, PM and/or luteolin diluted in HBSS were added for 30 min. The conversion of DCF-DA to DCF was monitored, and fluorescence was measured at an excitation and emission wavelength of 469 nm and 525 nm, respectively, using a cell imaging multimode reader (Biotek, VT, USA).

### GFP reporter gene assay

The pGF-AP-1-mCMV-EF1-Puro vector, pGF-NF-κB-mCMV-EF1-Puro vector, and pGF-MMP-1-mCMV-EF1-Puro vector, along with the packaging vectors (psPAX and pMD2.0G), were transfected into HEK293T cells using jetPEI transfection reagent according to the manufacturer’s instructions. Virus particles were prepared using a 0.45 μm syringe filter, and HaCaT cells were infected overnight with 8 µg/mL polybrene (EMD Millipore, Burlington, MA, USA). The cell culture medium was replaced with fresh growth medium, and the cells were cultured for 24 h. Transfected cells were selected by incubation in medium containing 2 mg/mL puromycin (Sigma, MO, USA) for 36 h. Transfected cells were cultured in 96-well black plates and incubated for 24 h. After incubation, the cells were starved in serum-free medium for 24 h, treated with luteolin (100, 200, or 400 nM) for 1 h, and then treated with PM. GFP fluorescence was monitored and measured at an excitation and emission wavelength of 469 nm and 525 nm, respectively, using a cell imaging multimode reader (Biotek, VT, USA).

### MKK4 kinase assay

The MKK4 kinase assay kit was purchased from Promega (Madison, WI, USA) and experiments were conducted according to the manufacturer’s instructions. Active MKK4 kinases were mixed with luteolin (100, 200, and 400 nM) in 1× kinase buffer (5% DMSO) and incubated with 400 nM substrate (JNK1) for 10 min, followed by incubation with ultra-pure ATP (10 µM) for 1 h. Subsequently, 5 µL of ADP-Glo™ Reagent was added and incubated for 40 min. Then, 10 µL of Kinase Detection Reagent was added and incubated for an additional 30 min. The luminescence was measured using a luminescence reader (BioTek, VT, USA).

### Pulldown assay

The pull-down assay was performed according to the method^25^. Luteolin–Sepharose 4 B beads were prepared by activating Sepharose 4 B powder with 1 mM HCl, followed by mixing luteolin with the coupling solution (0.1 M NaHCO_3_ and 0.5 M NaCl) and shaking at 4 °C overnight. For the pulldown assay, active MKK4 protein and HaCaT cell lysate were incubated with Luteolin–Sepharose 4B (or Sepharose 4B alone as a control) beads in reaction buffer [50 mmol/L Tris (pH 7.5), 5 mmol/L EDTA, 150 mmol/L NaCl, 1 mmol/L DTT, 0.01% Nonidet P-40, 2 µg/mL bovine serum albumin, and protease/phosphatase inhibitor] and mixed on a shaker at 4 °C for overnight. Beads were washed five times with reaction buffer.Proteins bound to the beads were analyzed using western blotting.

### Statistical analysis

All experiments were replicated three times, and the data were analyzed using the SPSS Statistics software (version 21.0; IBM, New York, NY, USA). To compare the differences between groups, one-way ANOVA was performed, followed by post hoc analysis using Duncan’s multirange test. Statistical significance was set at *P* < 0.05.

## Results

### Effects of Luteolin in PM-Induced MMP-1 in HaCaT keratinocytes

Previous studies have reported that MMP-1 expression is closely associated with skin aging. Therefore, we investigated the effects of luteolin on PM-induced MMP-1 expression. PM exposure increased MMP-1 production in HaCaT cells, and luteolin effectively inhibited PM-induced MMP-1 production (Fig. 1B, C and D) without affecting cell viability (Fig. 1F). Additionally, luteolin successfully suppressed PM-induced MMP-1 mRNA expression (Fig. 1E). These results indicate that luteolin can effectively inhibit PM-induced MMP-1 expression in HaCaT cells and suggest that skin aging caused by PM could be suppressed.

### Effects of Luteolin in PM-Induced skin inflammation

**Fig. 2:**
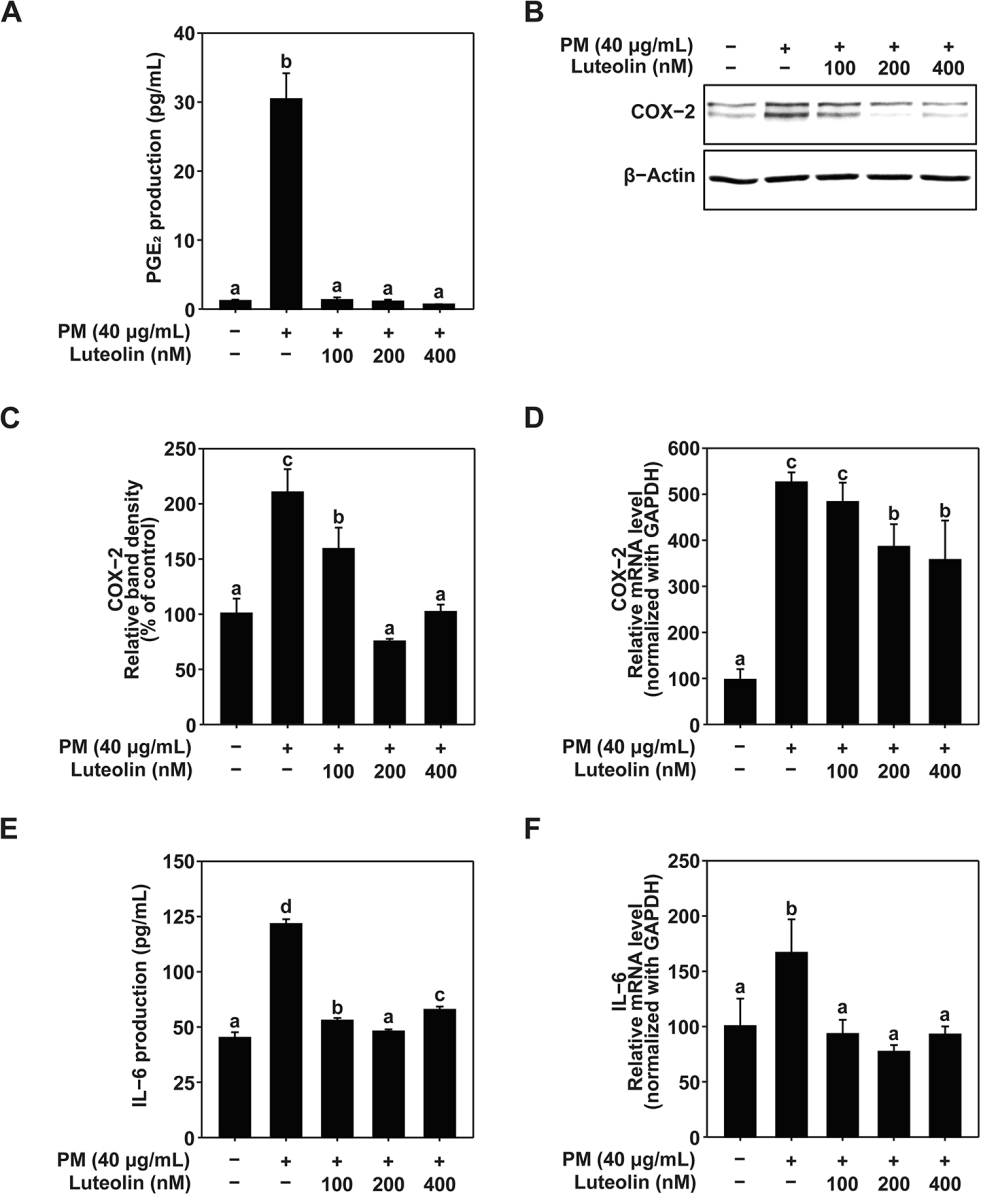
Effect of luteolin on PM-induced COX-2 and IL-6 production and expression. (**A**) Effect of luteolin on prostaglandin E_2_ (PGE2) production in HaCaT cells. HaCaT cells were pretreated with luteolin at the indicated concentrations for 1 h, followed by exposure to PM (40 µg/mL). After 24 h, the culture media were collected, and PGE2 production was measured using PGE2 enzyme immunoassay kits. (**B**, **C**) Effect of luteolin on COX-2 expression in HaCaT cells. HaCaT cells were pretreated with luteolin at the indicated concentrations for 1 h, followed by exposure to PM (40 µg/mL). After 24 h, the cells were lysed as described in the Materials and Methods. COX-2 protein levels were analyzed using western blotting. β-Actin was detected to verify equal loading of proteins. Images were captured using FUSION Solo S (VILVER Lourmat., Paris, FRA). (**D**) Effect of luteolin on COX-2 mRNA expression in HaCaT cells. COX-2 mRNA levels were analyzed using quantitative real-time PCR. HaCaT cells were pretreated with luteolin at the indicated concentrations for 1 h, followed by exposure to PM (40 µg/mL). After 24 h, the cells were lysed as described in the Materials and Methods section. (**E**) Effect of luteolin on IL-6 production in HaCaT cells. HaCaT cells were pretreated with luteolin at the indicated concentrations for 1 h, followed by exposure to PM (40 µg/mL). After 24 h, the culture media were collected, and IL-6 production was measured using ELISA assay kits. (**F**) Effect of luteolin on IL-6 mRNA expression in HaCaT cells. IL-6 mRNA levels were analyzed using quantitative real-time PCR. HaCaT cells were pretreated with luteolin at the indicated concentrations for 1 h, followed by exposure to PM (40 µg/mL). After 6 h, the cells were lysed as described in the Materials and Methods section. Data (*n* = 3) are shown as the mean ± SD. The mean with letters (a–d) on the graph indicates a significant difference (*p* < 0.05) in one-way ANOVA, and Duncan’s Multiple Range test was used as a post-test.

IL-6 is a soluble cytokine with diverse effects on inflammation and immune system. Although its production is carefully regulated, dysregulated IL-6 expression can contribute to the pathogenesis of chronic inflammation and autoimmune diseases^31^. Overexpression of COX-2 and IL-6 is closely associated with inflammatory responses in the skin, and exposure to PM-induces induces overexpression of COX-2 and IL-6^32^. Therefore, we investigated the effect of luteolin on PM-induced expression of COX-2 and IL-6. PM exposure increased PGE_2_ production, whereas luteolin effectively inhibited PM-induced PGE_2_ production (Fig. 2A). To identify mechanisms underlying PGE_2_, COX-2 expression was investigated. Luteolin treatment downregulated PM-induced COX-2 expression (Fig. 2B and C). The mRNA expression of COX-2 was also inhibited by luteolin (Fig. 2D). Moreover, PM-induced IL-6 production and mRNA expression was suppressed by luteolin (Fig. 2E and F). These results indicate that luteolin can effectively inhibit PM-induced COX-2 and IL-6 expression in HaCaT cells, suggesting that skin inflammation caused by PM can be suppressed.

### Effects of Luteolin in PM-Induced AP-1 and NF-κB transactivation in HaCaT cell

**Fig. 3:**
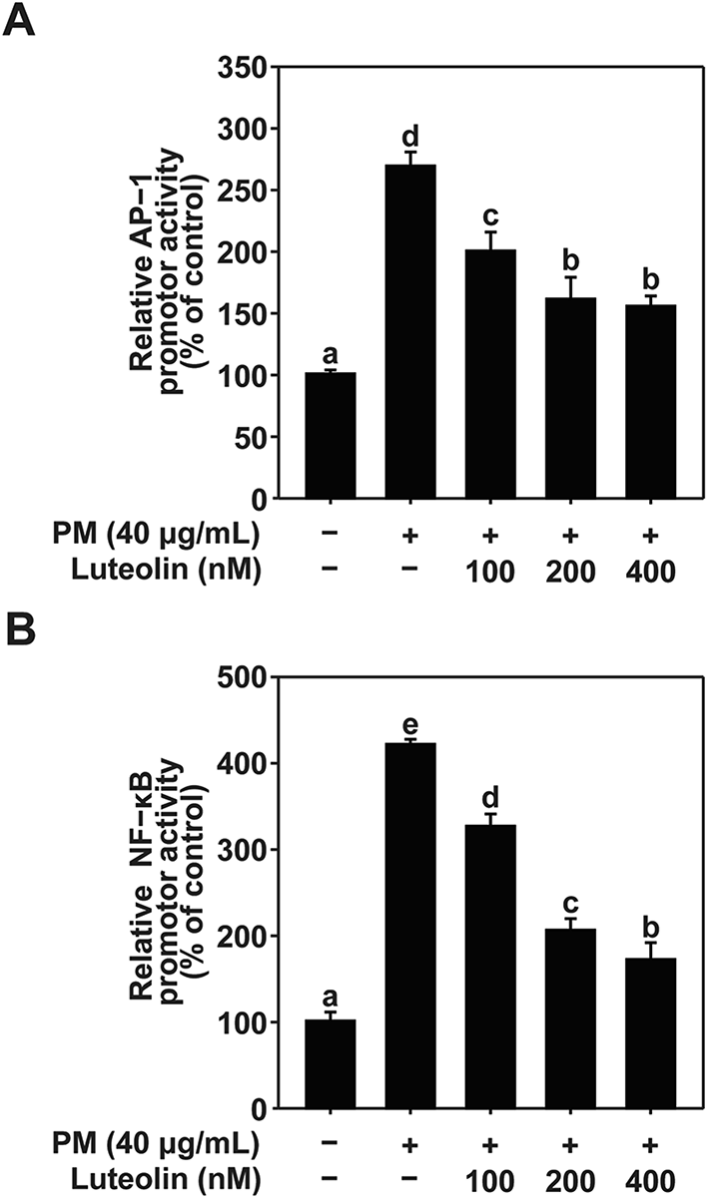
Effect of luteolin on PM-induced AP-1 and NF-κB activity in HaCaT cells. Transactivation of (**A**) AP-1 and (**B**) NF-κB was measured using a GFP reporter gene assay. HaCaT cells transduced with AP-1 and NF-κB were pretreated with luteolin at the indicated concentrations for 1 h, followed by exposure to PM (40 µg/mL). After 24 h, transactivation was measured at excitation and emission wavelengths of 469 and 525 nm, respectively (GFP). Data (*n* = 3) are shown as the mean ± SD. Means with letters (a–e) on the graph indicate a significant difference (*p* < 0.05) in a one-way ANOVA, and Duncan’s Multiple Range test was used as a post-hoc test.

PM exposure induces the activation of AP-1 and NF-κB. AP-1 is a crucial transcription factor for MMP-1 expression, whereas NF-κB is implicated in the expression of COX-2 and IL-6 ^33,34^. Therefore, we examined the effect of luteolin on NF-κB and AP-1 transactivation using HaCaT cells stably transfected with NF-κB or AP-1 luciferase reporter plasmids. Based on the results of the GFP Reporter Gene Assay, luteolin dose-dependently downregulated the PM-induced transactivation of AP-1 (Fig. 3A) and NF-κB (Fig. 3B). Our results suggest that luteolin inhibits PM-induced MMP-1, COX-2, and IL-6 expression by suppressing AP-1 and NF-κB activation.

### Luteolin inhibits phosphorylation of JNK1/2, p38, but increases phosphorylation of MKK4

**Fig. 4:**
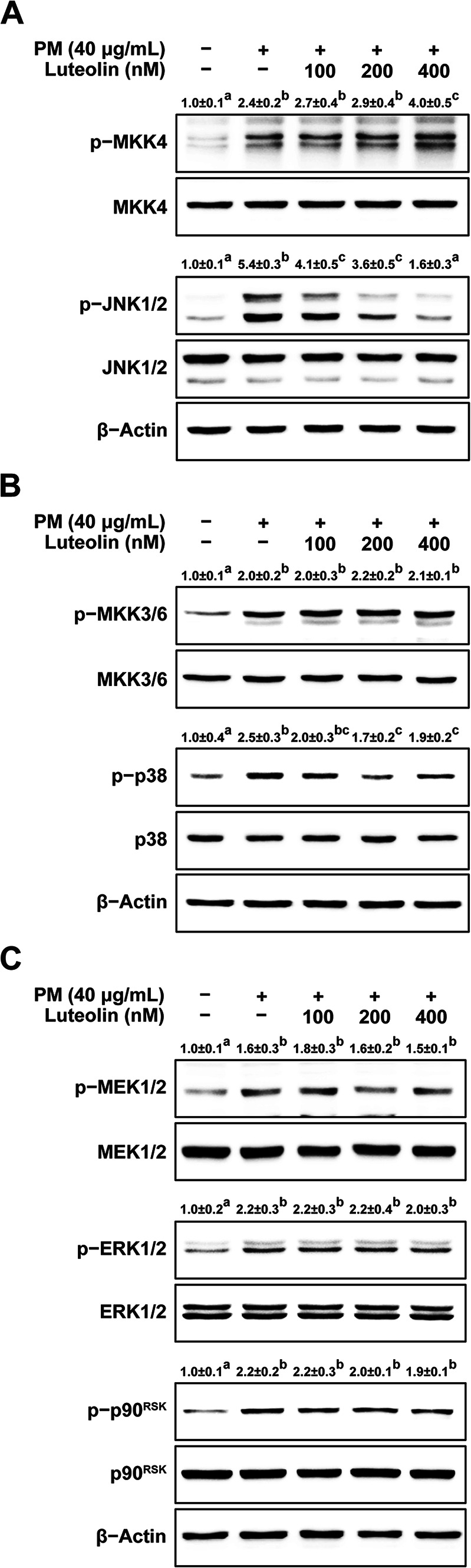
Effect of luteolin on PM-induced MAPK signaling pathways in HaCaT cells. Luteolin reduced PM-induced phosphorylation of (**A**) MKK4-JNK1/2, (**B**) MKK3/6-p38, and (**C**) MEK1/2-ERK1/2-p90RSK. HaCaT cells were pretreated with luteolin at the indicated concentrations for 1 h, followed by exposure to PM (40 µg/mL). After 2 h, the cells were lysed as described in the Materials and Methods section. Phosphorylated and total protein levels were analyzed by western blotting. Images were captured using FUSION Solo S software (VILVER Lourmat, Paris, FRA). Data (*n* = 3) are shown as the mean ± SD. Means with letters (a–c) on the graph indicate a significant difference (*p* < 0.05) in a one-way ANOVA, and Duncan’s Multiple Range test was used as a post-hoc test.

The MAPK signaling pathway regulates the activity of AP-1 and NF- κB^35^. Therefore, the effect of luteolin on PM-induced phosphorylation of MAP kinases was investigated. Luteolin treatment downregulated the PM-induced JNK1/2 phosphorylation in a dose-dependent manner (Fig. 4A). Additionally, PM-induced p38 phosphorylation was inhibited (Fig. 4B). However, the PM-induced phosphorylation of MKK3/6 and MEK1/2-ERK1/2-p90^RSK^ did not change after luteolin treatment (Fig. 4B and C). Even phosphorylation of MKK4 increased after treatment with 400 nM luteolin (Fig. 4A). The mitogen-activated protein kinase (MAPK) signaling pathway comprises three primary axes: JNK, p38, and ERK pathways^38^. Each pathway plays distinct roles in cellular responses to stress and inflammation. JNK and p38 are activated by stress stimuli such as oxidative stress and inflammatory cytokines, while ERK primarily responds to growth factors and mitogens^39^. MKK4, as an upstream kinase, selectively phosphorylates JNK and, to a lesser extent, p38, thereby modulating downstream signaling events critical for inflammatory and stress responses. Based on these observations, our results suggest that luteolin specifically affects the activity of MKK4.These results suggest that luteolin affects the activity of MKK4, leading to the decreased phosphorylation of JNK1/2 and p38.

### Luteolin inhibits MKK4 activity though direct binding

**Fig. 5:**
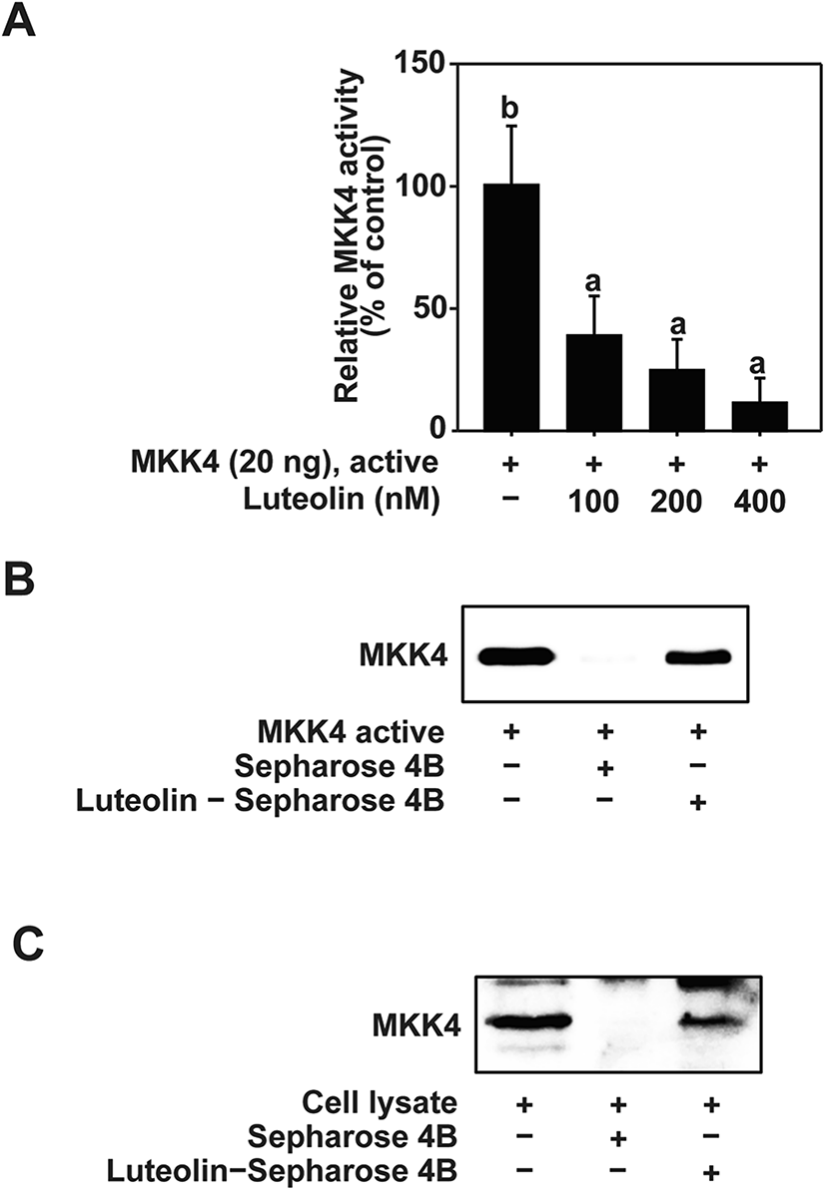
Effects of luteolin on MKK4 activity with direct binding. (**A**) Luteolin inhibits MKK4 activity. (**B**) Binding of luteolin to active MKK4. Luteolin–MKK4 binding was investigated using western blotting using an antibody against MKK4: Lane 1 (input control), MKK4 (20 ng), protein standard. Lane 2 (control), Sepharose 4B beads. Lane 3, MKK4 was pulled down using Luteolin–Sepharose 4B beads. (**C**) Binding of luteolin to MKK4 in HaCaT cell lysate. Luteolin–MKK4 binding was confirmed by Western blot using an antibody against MKK4: Lane 1 (input control), HaCaT cell lysate (0.2 µg), protein standard. Lane 2 (control), Sepharose 4B beads. Lane 3, MKK4 was pulled down using Luteolin–Sepharose 4B beads. Data (*n* = 3) are shown as the mean ± SD. The mean with letters (a-b) on the graph indicates a significant difference (*p* < 0.05) in one-way ANOVA, and Duncan’s Multiple Range test was used as a post-test.

Luteolin inhibits JNK1/2 and p38 phosphorylation However, this paradoxically increased the phosphorylation of MKK4. Therefore, we hypothesized that luteolin may affect the activity of MKK4 and subsequently investigated the effect of luteolin on MKK4. The kinase assay results showed that luteolin effectively inhibited the activity of MKK4 (Fig. 5A). To elucidate the mechanism by which luteolin modulates MKK4 activity, we investigated whether luteolin binds directly to MKK4. Based on the results of the pull-down assay, we found that luteolin binds to both recombinant active and HaCaT cell lysate MKK4 (Fig. 5B and C).

### Effects of Luteolin in PM-Induced ROS production in HaCaT cells

**Fig. 6:**
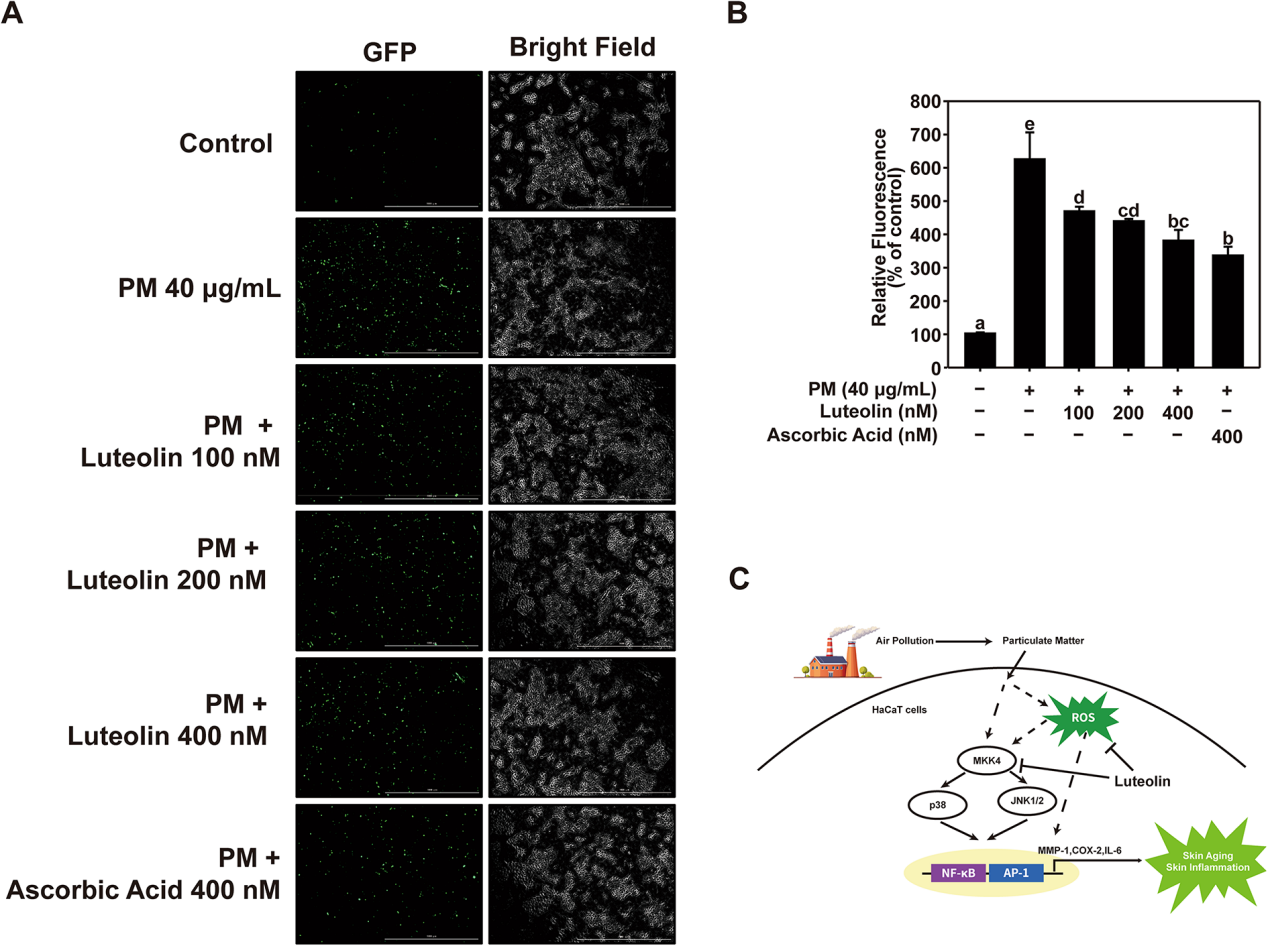
Luteolin reduces PM-Induced ROS production in HaCaT cells. (**A**, **B**) Images and quantification of the effects of luteolin on PM-induced ROS reduction, using a DCF-DA fluorescence-based assay. Data (*n* = 3) are shown as the mean ± SD. Means with letters (a-e) on the graph indicate a significant difference (*p* < 0.05) in a one-way ANOVA, and Duncan’s Multiple Range test was used as a post-hoc test. (**C**) Schematic representation of the proposed molecular mechanism underlying luteolin’s protective effects against particulate matter-induced MMP-1 expression and inflammatory responses in human keratinocytes.

The ROS generated by PM play a pivotal role in activating the MAPK signaling pathway^40^. Therefore, we investigated the effect of luteolin on PM-induced intracellular ROS production. Luteolin treatment downregulated PM-induced ROS production in HaCaT cells in a dose-dependent manner (Fig. 6A and B). These results suggest that luteolin effectively suppresses ROS and revealed that luteolin is an effective antioxidant that mitigates oxidative stress induced by PM.

## Discussion

PM is a mediator of skin health deterioration, along with UV radiation, which has traditionally been recognized as a major cause^42^. Furthermore, when the skin is exposed to both PM and UV radiation, combined adverse effects can occur^32^. With the emergence of these studies, natural compounds that suppress the adverse effects of PM have attracted attention^43^. Natural compounds have been traditionally used in skin care for centuries, and their beneficial activities are considered potential natural sources for maintaining skin health and beauty^44^. Therefore, this study aimed to investigate the protective effects of luteolin against PM-induced skin aging and inflammation.

We focused on MMP-1, COX-2, and IL-6 to elucidate the molecular mechanisms by which luteolin mitigates PM-induced skin aging and inflammation. These markers were chosen because of their crucial roles in the inflammatory response, skin aging, and tissue remodeling. MMP-1 degrades collagen, a key structural protein in the skin, thereby accelerating skin aging and exacerbating dermal damage. COX-2, a proinflammatory enzyme, catalyzes the production of prostaglandins and amplifies the inflammatory cascade. IL-6, a potent cytokine that promotes inflammation, has been implicated in various dermatological pathologies. By investigating the effects of luteolin on these specific markers, we aimed to demonstrate its potential as an anti-inflammatory and anti-aging agent capable of protecting against PM-induced skin damage by inhibiting critical inflammatory pathways and mediators.

MKK4, a member of the MAPK kinase family, consists of 399 amino acids and contains 11 subdomains that fold into two distinct lobes: a larger, primarily α-helical C-terminal lobe connected by a flexible hinge region, and a smaller, N-terminal lobe composed of five β-sheets and one α-helix^45^. Upon activation, MKK4 undergoes dual phosphorylation at Ser257 and Thr261, located in the activation loop. Subsequently, activated MKK4 phosphorylates and activates both JNK and p38 kinases through dual phosphorylation of Thr and Tyr residues within the Thr-Pro-Tyr and Thr-Gly-Tyr motifs in their respective activation loops^46^. Notably, MKK4 has a more pronounced effect on JNK activation than on p38^45^. The inhibition of MKK4 expression prevents skin cancer, as reported previously^47,48^. In this study, we observed that the inhibitory effects of luteolin on PM-induced MMP-1, COX-2, and IL-6 expression appeared to occur through the direct inhibition of MKK4.

Assuming that the anti-aging and anti-inflammatory effects of luteolin occur through direct inhibition of MKK4, several supporting pieces of evidence have been reported. When examining the phosphorylation levels initially, it was observed that upon treatment with luteolin, the phosphorylation of JNK1/2 and p38 decreased, whereas there was no change in the phosphorylation levels of MKK3/6, an upstream regulator of p38. Furthermore, upon treatment with luteolin, the change in the phosphorylation levels of JNK1/2 was more pronounced than that of p38, which is consistent with the characteristics of MKK4. Moreover, treatment with 400 nM luteolin led to a significant increase in the phosphorylation levels of MKK4, indicating a potential feedback loop effect. In the feedback loop effect, pharmaceutical inhibitors increase the phosphorylation of target kinases through a feedback loop^49^. Based on these findings, we postulated that luteolin directly inhibits MKK4 activity. Using a kinase assay, we demonstrated that luteolin reduced MKK4 activity. Furthermore, in studies inhibiting MKK4 activity with the structurally similar flavonoids Myricetin, and Cyanidin^50,51^, the phosphorylation levels of MKK4-JNK1/2 were similar to those of luteolin. Pull-down assays were performed to investigate how luteolin regulates MKK4 activity. These results confirm the direct binding between luteolin and MKK4.

In this study, we demonstrated that luteolin directly inhibited MKK4, indicating that MKK4 is a specific molecular target of luteolin. However, luteolin also inhibits ROS production. To reconcile these findings, we proposed that luteolin operates via a dual mechanism. By directly inhibiting MKK4, luteolin modulates intracellular signaling pathways. Concurrently, its ability to suppress ROS production suggests an additional mechanism by which luteolin mitigates cellular oxidative stress within the cell. These dual actions of luteolin imply a multifaceted protective role, which potentially contributes to its therapeutic efficacy. These findings highlight the importance of considering both direct molecular targets and broader cellular effects when evaluating the potential of luteolin for therapeutic development.

In conclusion, this study demonstrated the potential anti-aging and anti-inflammatory effects of luteolin. The findings of this study highlight the potential of luteolin as a protective agent against particulate matter-induced skin damage. To translate these findings into practical applications, future research will focus on conducting clinical trials to validate the efficacy and safety of luteolin-based formulations in humans. These studies will provide critical insights into the effectiveness of luteolin in mitigating pollution-induced skin aging and inflammation in real-world conditions.

Additionally, the development of luteolin-containing cosmetic ingredients will be a key focus. Leveraging the mechanistic insights gained from this study, such formulations will be designed to counteract oxidative stress and inflammatory responses, addressing the growing consumer demand for anti-pollution skincare solutions. These efforts will bridge the gap between laboratory research and consumer applications, paving the way for innovative, science-backed cosmetic products.

## Electronic supplementary material

Below is the link to the electronic supplementary material.

Supplementary Material 1

## Data Availability

The datasets used and/or analysed during the current study available from the corresponding author on reasonable request.
