# Supplementary material for: Luteolin targets MKK4 to attenuate particulate matter-induced MMP-1 and inflammation in human keratinocytes
Source: Sci Rep. 2025 May 15;15:16848. doi: 10.1038/s41598-025-01090-3 (PMC12081746; doi:10.1038/s41598-025-01090-3)
Supplement: Supplementary file 1 — Supplementary Material 1 [file 41598_2025_1090_MOESM1_ESM.pdf]

Figure 1. Effect of luteolin on PM-induced MMP-1 production and expression.

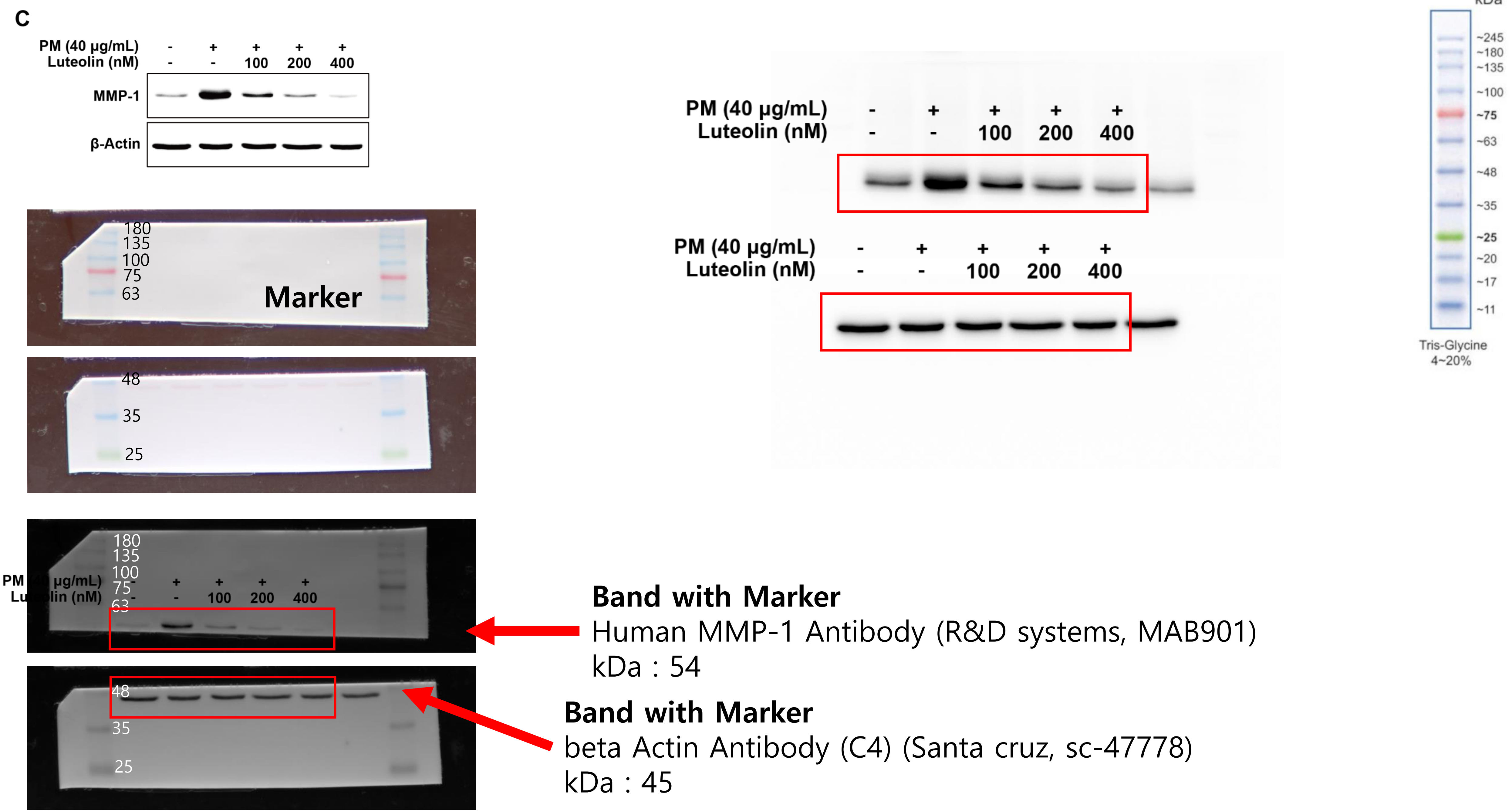

Figure 2. Effect of luteolin on PM-induced COX-2 and IL-6 production and expression.

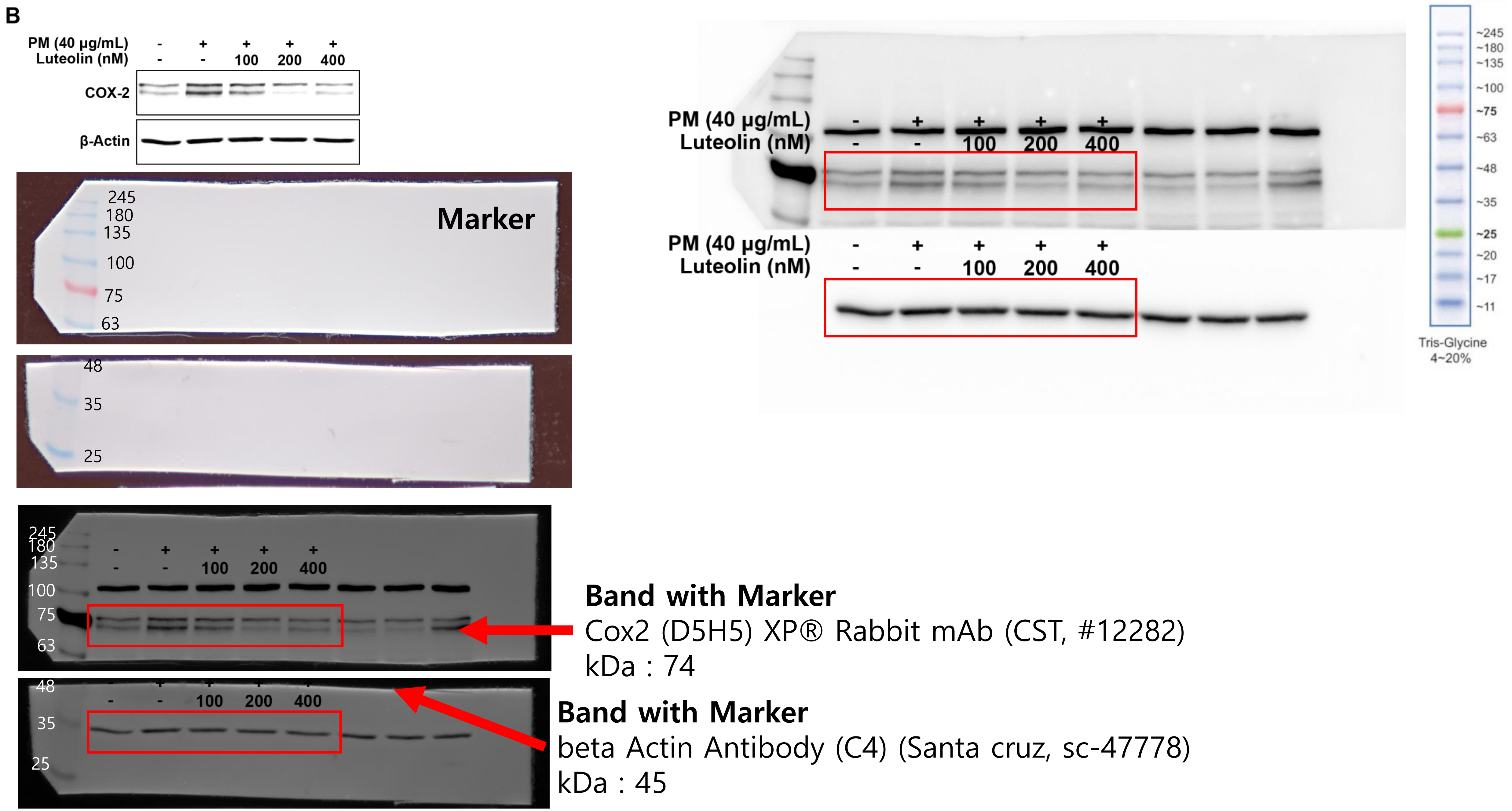

Figure 4. Effect of luteolin on PM-induced MAPK signaling pathways in HaCaT cells.

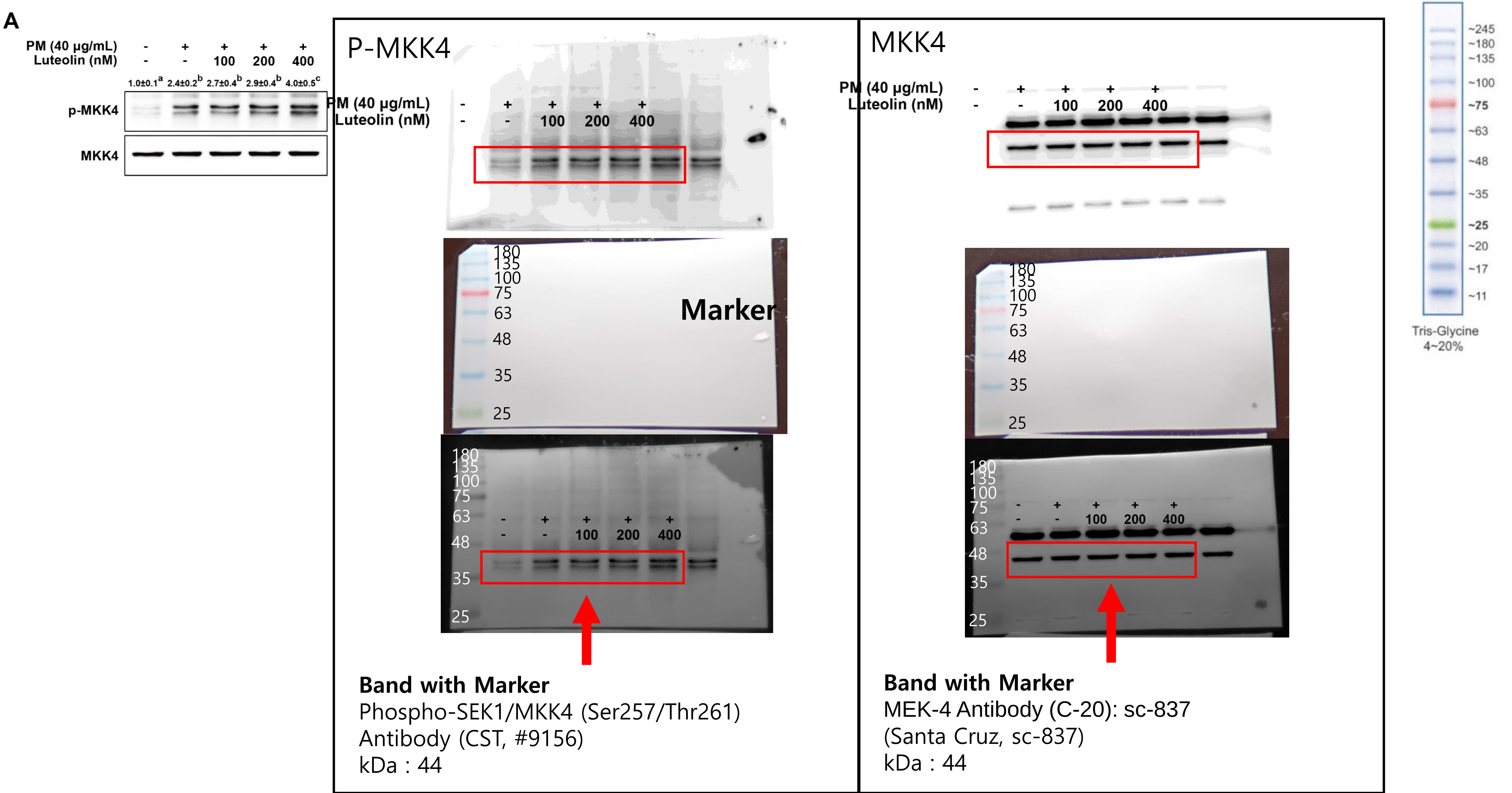

Figure 4. Effect of luteolin on PM-induced MAPK signaling pathways in HaCaT cells.

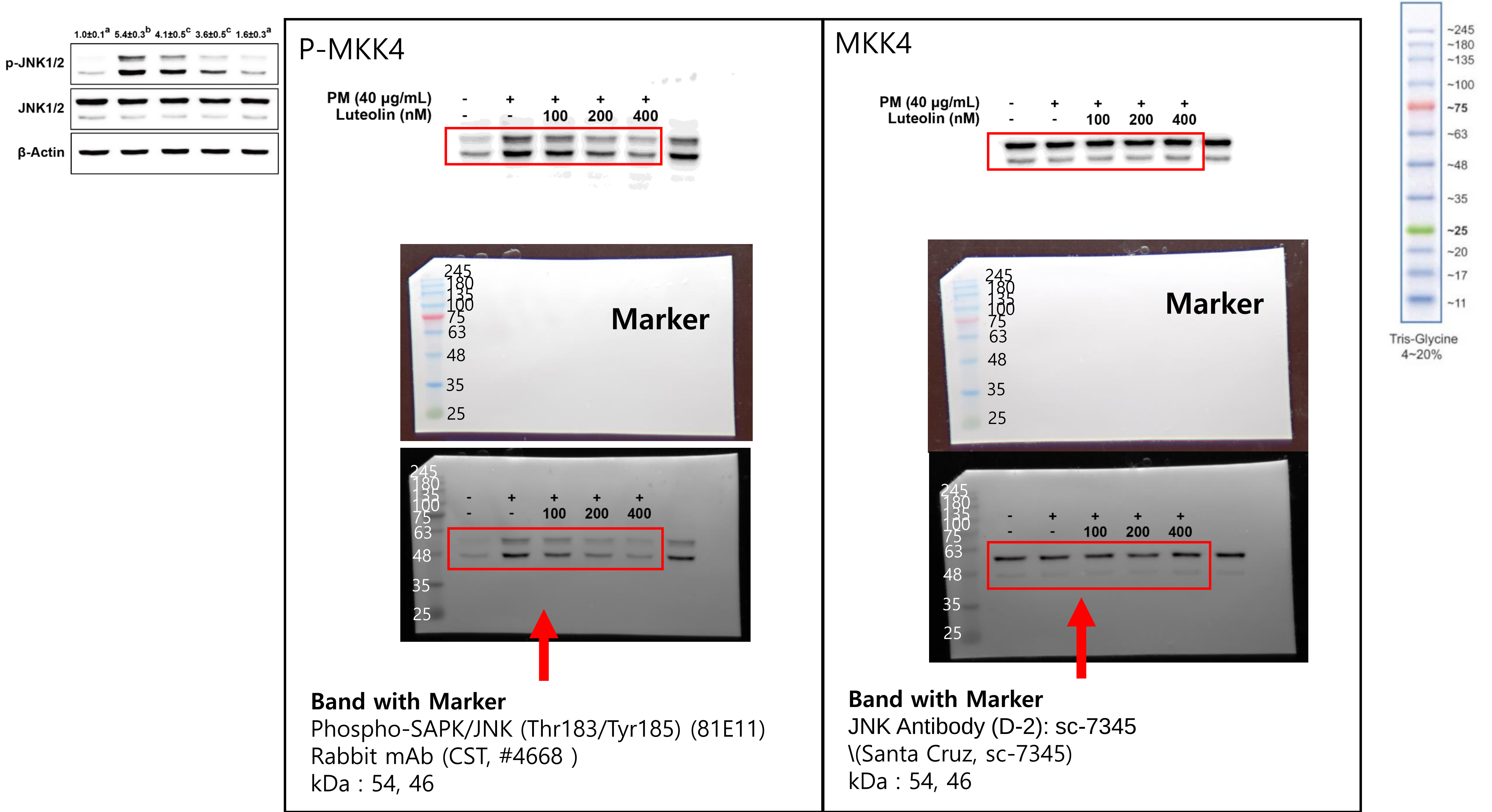

Figure 4. Effect of luteolin on PM-induced MAPK signaling pathways in HaCaT cells.

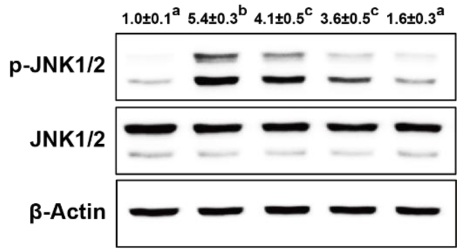

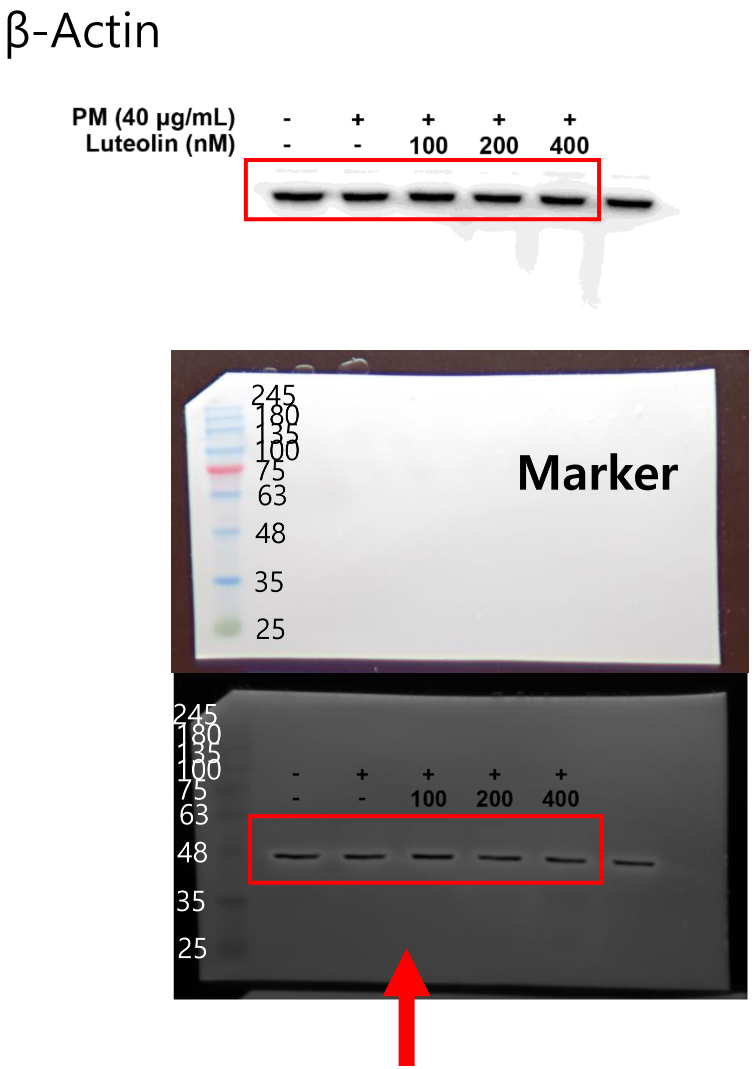

Band with Marker
beta Actin Antibody (C4) (Santa cruz, sc-47778)
kDa : 45

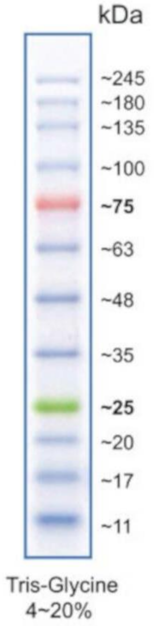

Figure 4. Effect of luteolin on PM-induced MAPK signaling pathways in HaCaT cells.

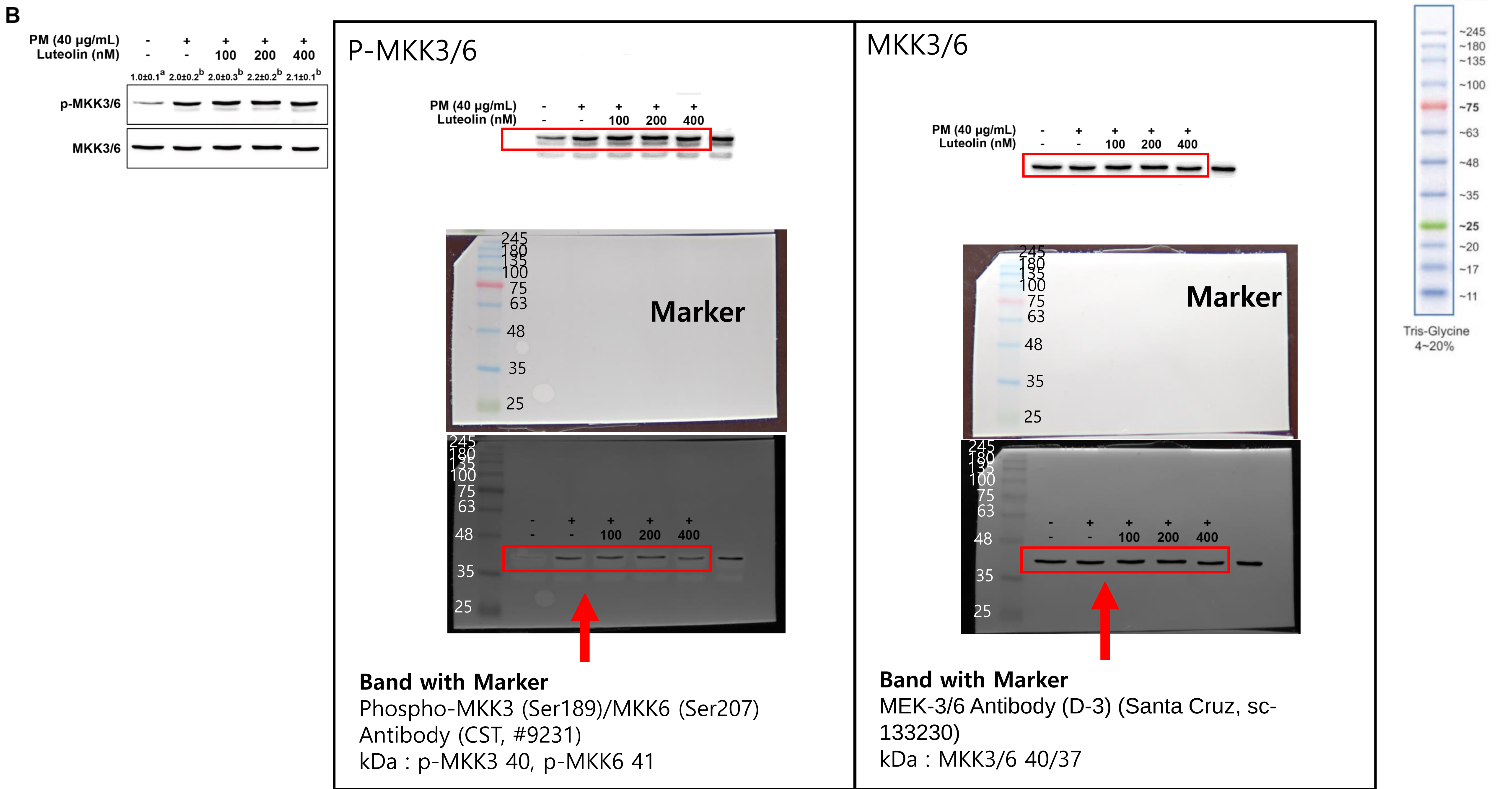

Figure 4. Effect of luteolin on PM-induced MAPK signaling pathways in HaCaT cells.

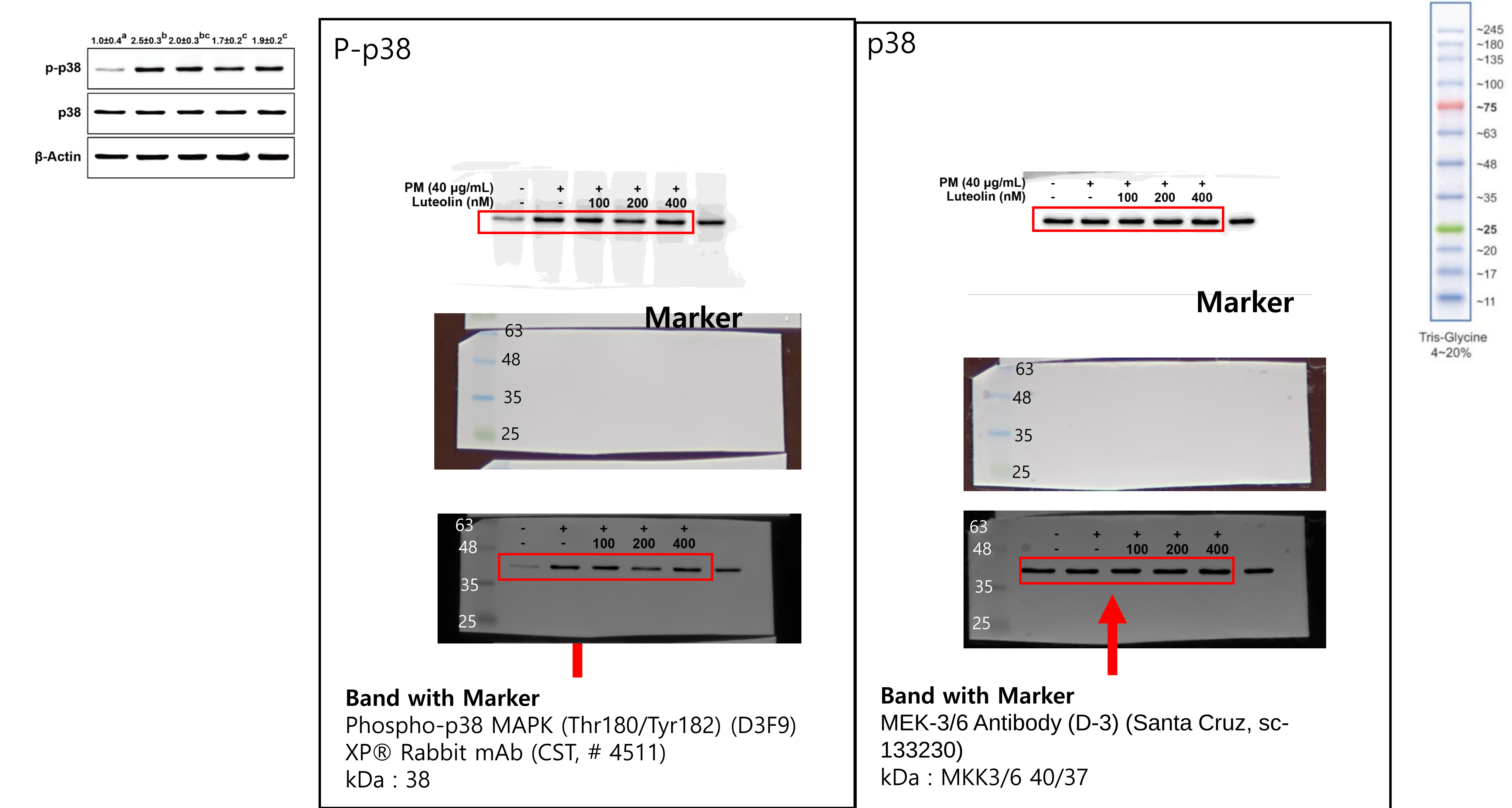

Figure 4. Effect of luteolin on PM-induced MAPK signaling pathways in HaCaT cells.

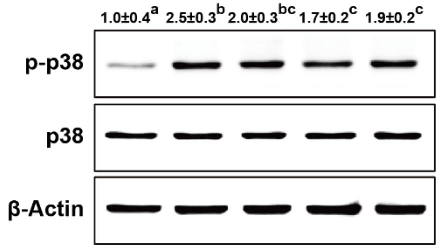

β-Actin

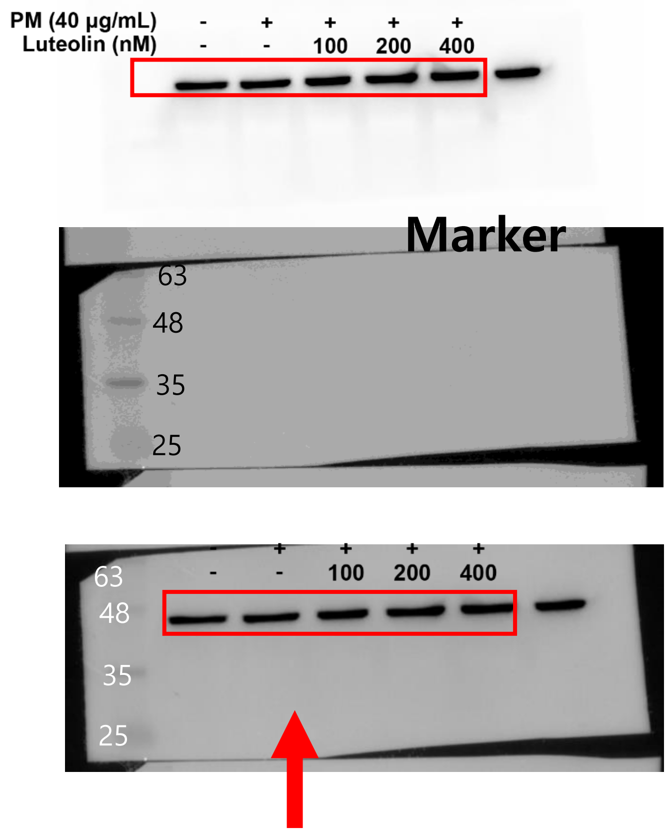

Band with Marker
beta Actin Antibody (C4) (Santa cruz, sc-47778)
kDa : 45

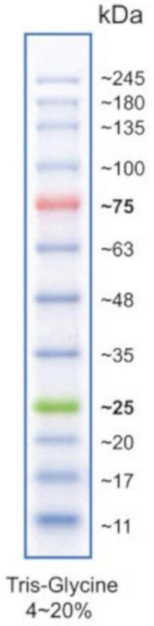

Figure 4. Effect of luteolin on PM-induced MAPK signaling pathways in HaCaT cells.

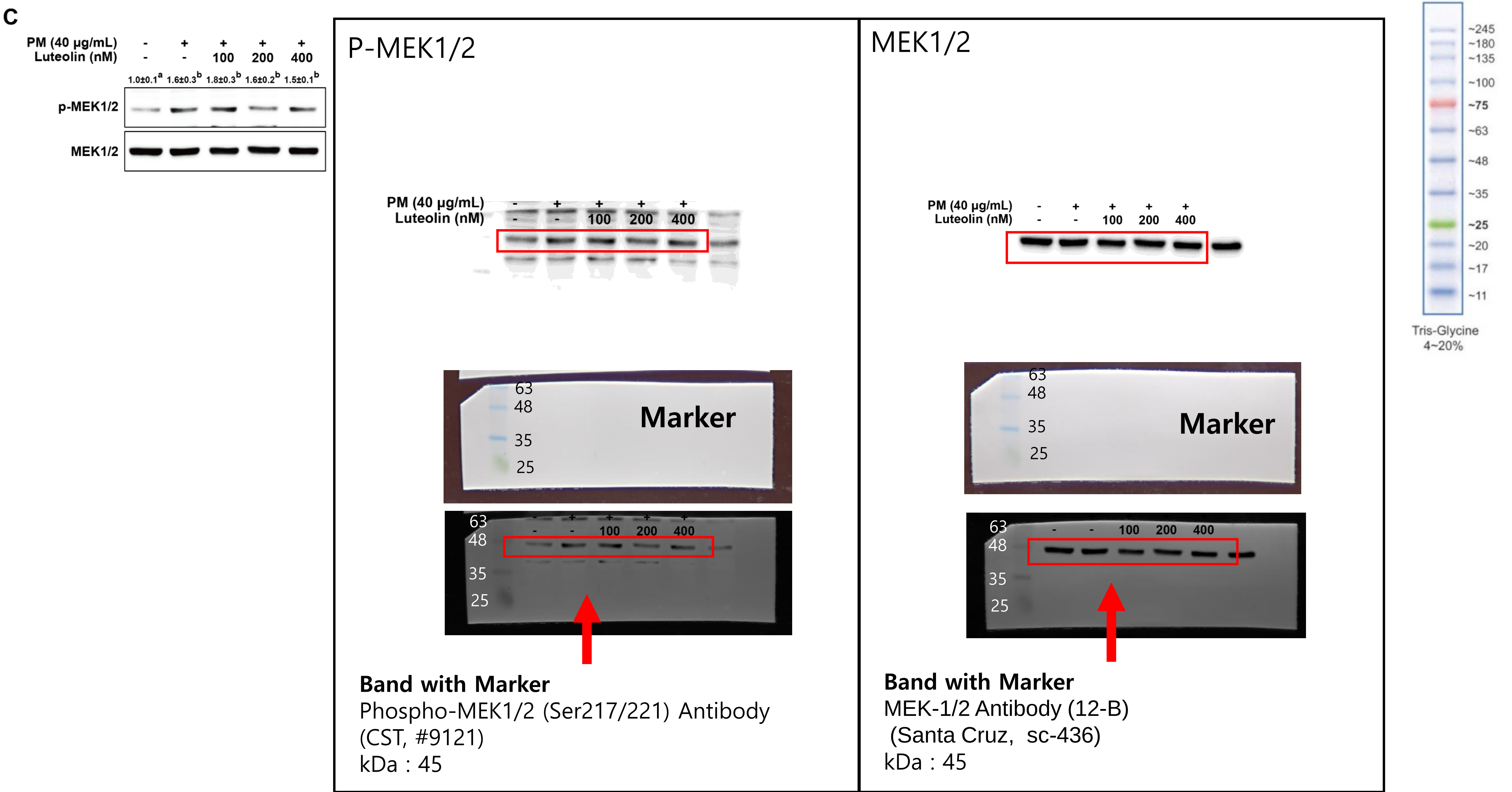

Figure 4. Effect of luteolin on PM-induced MAPK signaling pathways in HaCaT cells.

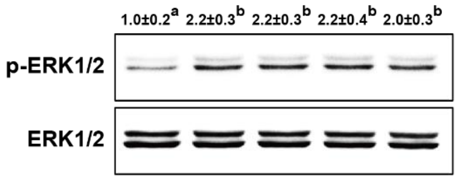

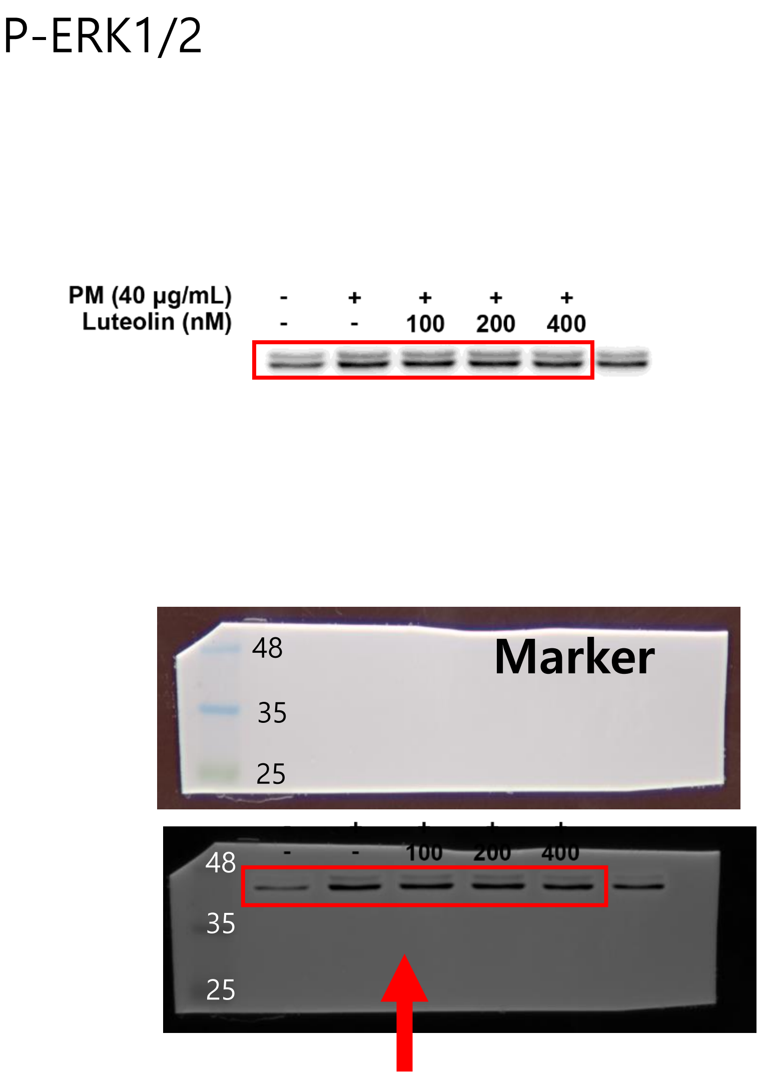

Band with Marker

Phospho-p44/42 MAPK (Erk1/2) (Thr202/Tyr204) Antibody (CST, #9101)

kDa : 42, 44

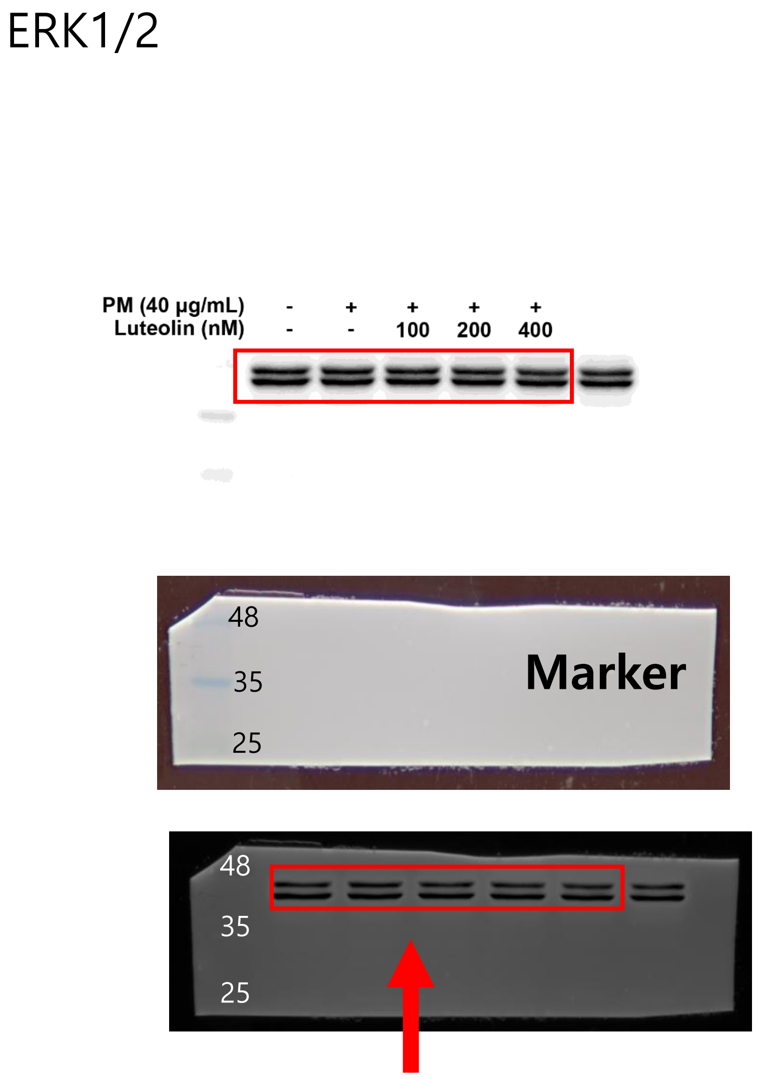

Band with Marker

ERK 1/2 Antibody (C-9): sc-514302 (Santa Cruz, sc-514302)

kDa : 42, 44

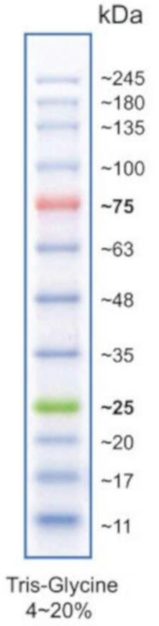

Figure 4. Effect of luteolin on PM-induced MAPK signaling pathways in HaCaT cells.

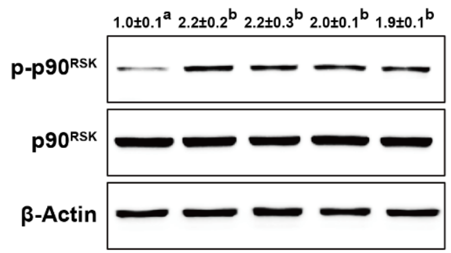

P-p90RSK

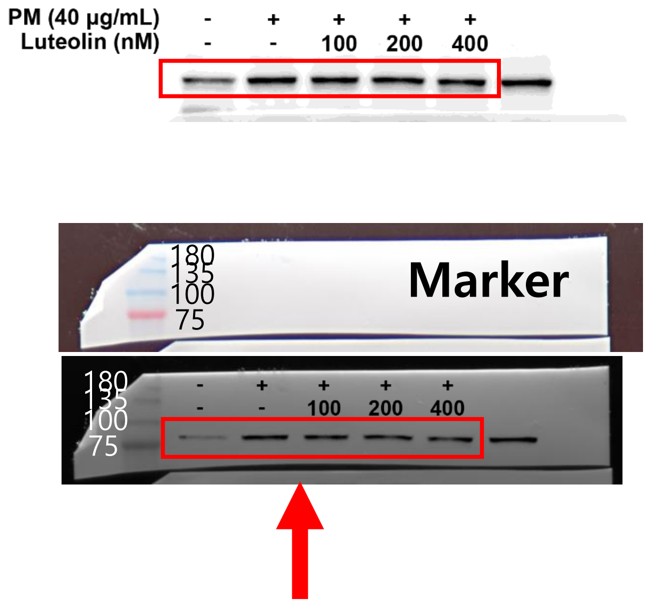

Band with Marker
Phospho-p90RSK (Thr359/Ser363) Antibody (CST, #9344)
kDa : 90

p90RSK

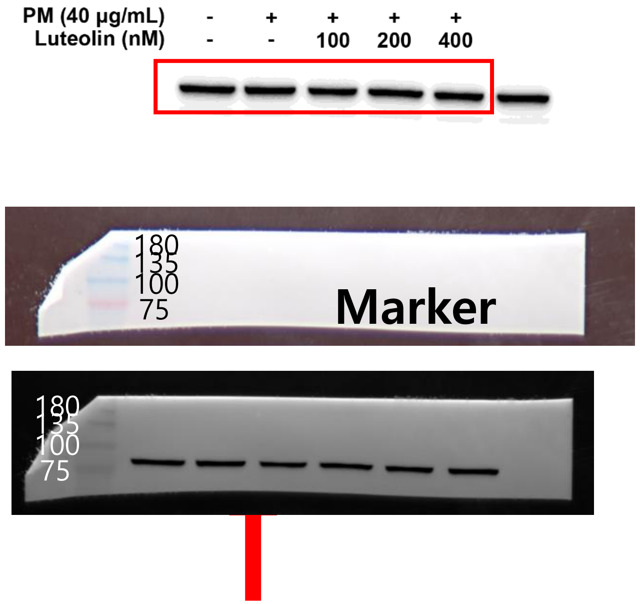

Band with Marker
Rsk-2 Antibody (E-1): (Santa Cruz, sc-9986)
kDa : 80

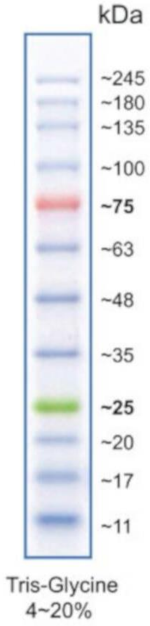

Fig 5. Effects of luteolin on MKK4 activity with direct binding.

B

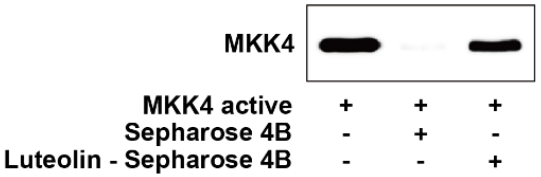

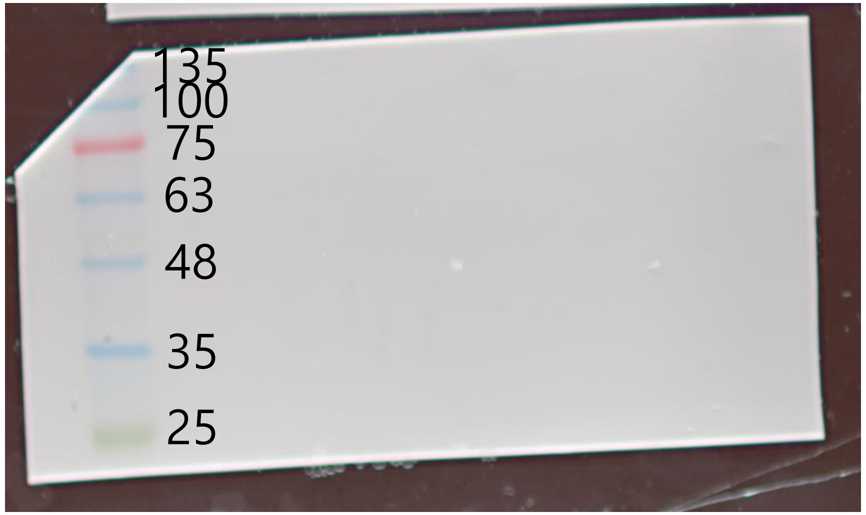

Marker

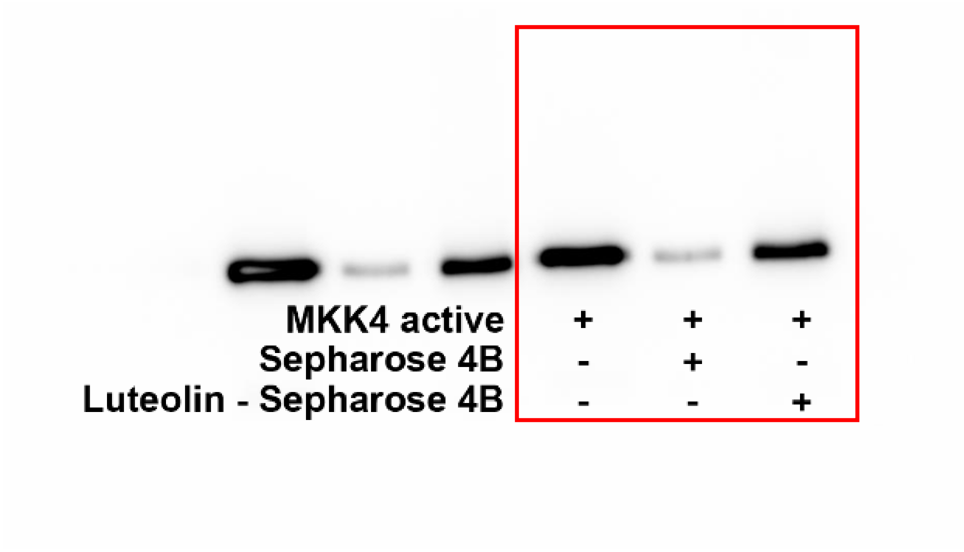

MKK4 Raw image

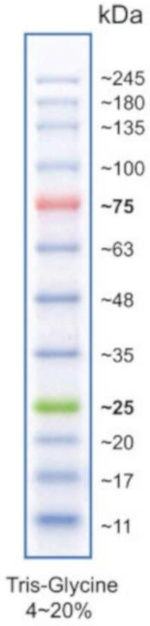

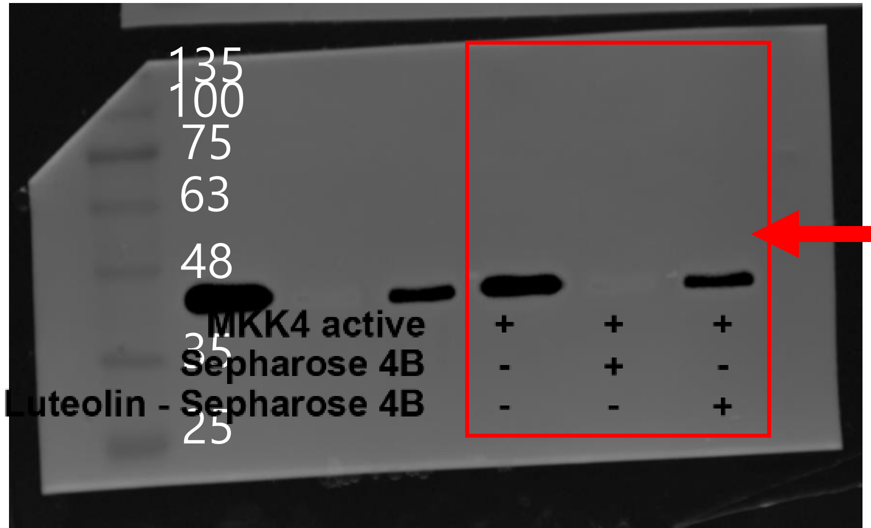

Band with Marker

SEK1/MKK4 Antibody (CST, #9152)

kDa : 44

Fig 5. Effects of luteolin on MKK4 activity with direct binding.

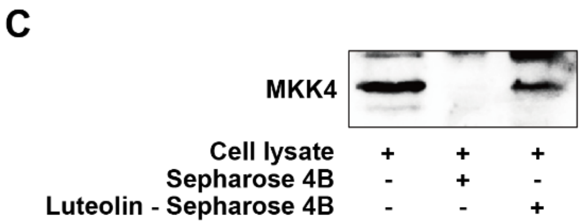

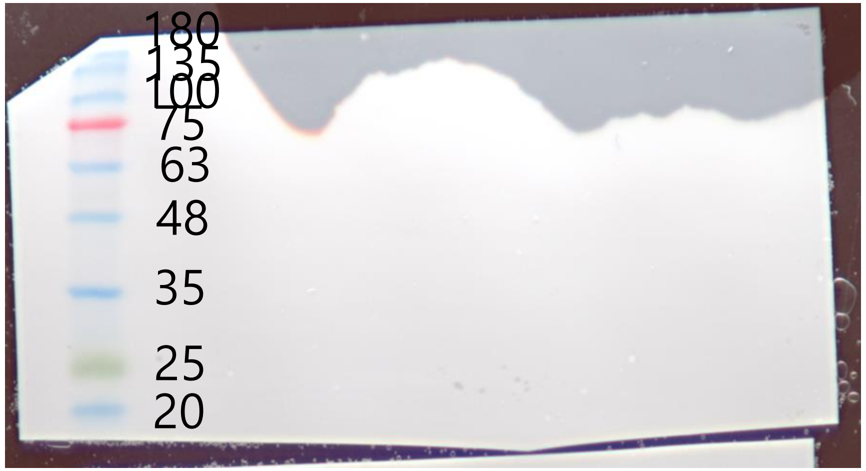

Marker

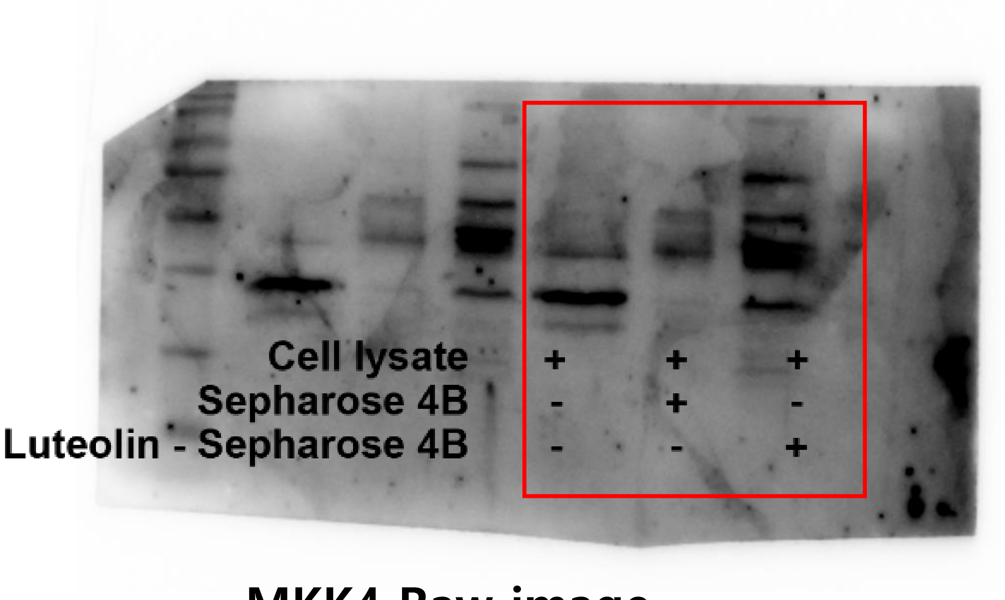

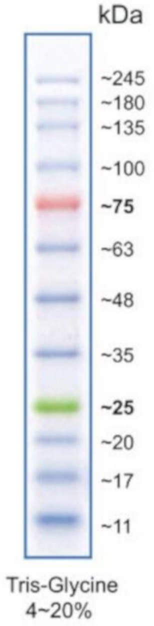

MKK4 Raw image

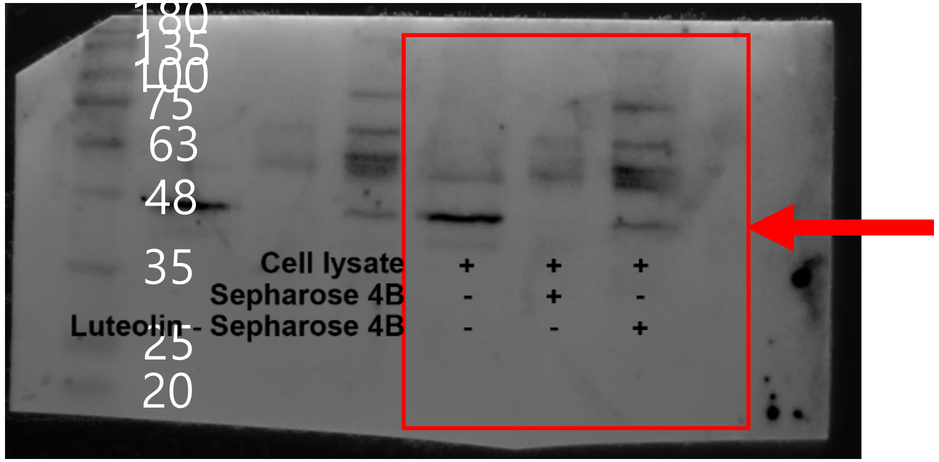

Band with Marker

SEK1/MKK4 Antibody (CST, #9152)

kDa : 44

Fig 5. Effects of luteolin on MKK4 activity with direct binding.

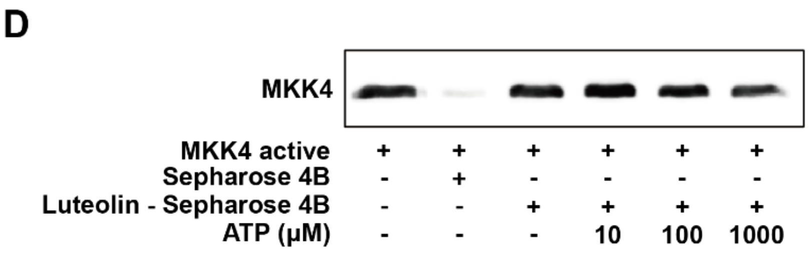

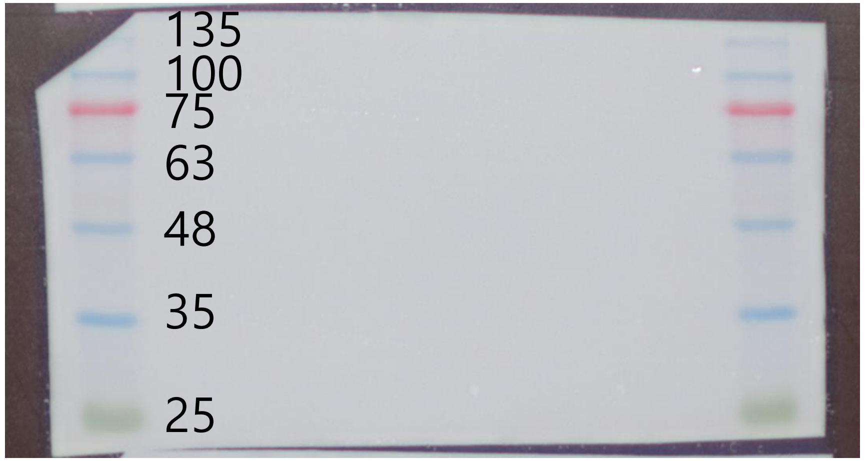

Marker

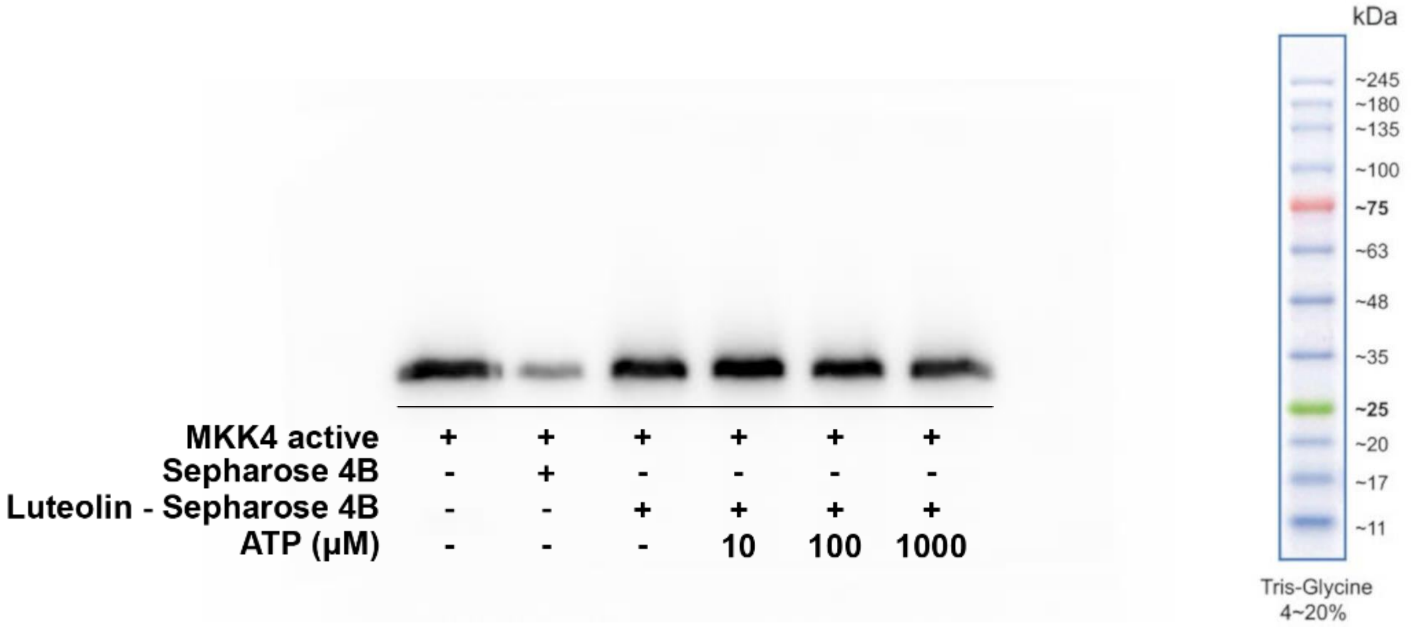

MKK4 Raw image

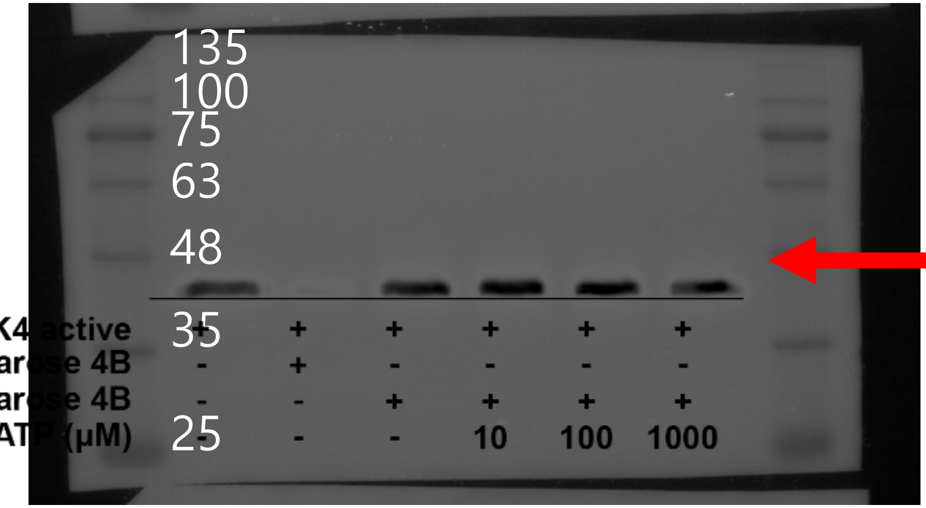

Band with Marker
SEK1/MKK4 Antibody (CST, #9152)
kDa : 44
